# Randomised Phase II Trial (NCT00637975) Evaluating Activity and Toxicity of Two Different Escalating Strategies for Pregabalin and Oxycodone Combination Therapy for Neuropathic Pain in Cancer Patients

**DOI:** 10.1371/journal.pone.0059981

**Published:** 2013-04-05

**Authors:** Marina Chiara Garassino, Sheila Piva, Nicla La Verde, Ilaria Spagnoletti, Vittorio Iorno, Claudia Carbone, Antonio Febbraro, Anna Bianchi, Annalisa Bramati, Anna Moretti, Monica Ganzinelli, Mirko Marabese, Marta Gentili, Valter Torri, Gabriella Farina

**Affiliations:** 1 Medical Oncology Unit 1, Fondazione IRCCS Istituto Nazionale dei Tumori, Milan, Italy; 2 Department of Oncology, A.O. Fatebenefratelli & Oftalmico, 23, Milan, Italy; 3 A.O. Sacro Cuore di Gesù, Fatebenefratelli, Benevento, Italy; 4 Policlinico Maggiore, Milan, Italy; 5 Department of Oncology Ospedale Serbelloni, Gorgonzola, Italy; 6 Department of Neurology A.O. Sant’Antonio Abate, Gallarate, Italy; 7 Department of Oncology “Mario Negri” Institute, Milan, Italy; The James Cook University Hospital, United Kingdom

## Abstract

**Purpose:**

Neuropathic pain is commonly associated with cancer. Current treatments include combination opioid and adjuvant therapies, but no guidelines are available for dose escalation strategies. This phase II study compared the efficacy and tolerability of two dose escalation strategies for oxycodone and pregabalin combination therapy.

**Methods:**

Patients (N = 75) with oncological neuropathic pain, previously untreated with pregabalin, were recruited in 5 Italian institutions between 2007 and 2010. Patients were randomised to two different dose escalation strategies (arm A; N = 38) oxycodone at a fixed dose with increasing pregabalin doses; (arm B; N = 37) pregabalin at a fixed dose with increasing oxycodone doses. Patients were evaluated from daily diaries and follow-ups at 3, 7, 10, and 14 days after beginning treatment with a numerical rating scale (NRS), neuropathic pain scale (SDN), and well-being scale (ESAS). The primary endpoint was a ≥1/3 reduction in pain (NRS); secondary endpoints included the time to analgesia and adverse effects. The study had a 90% probability of detecting the best strategy for a true difference of at least 15%.

**Results:**

More patients in arm A (76%) than arm B (64%) achieved ≥1/3 overall pain reduction even after controlling for baseline factors (gender, baseline pain). Group A reported fewer side effects than group B; constipation 52.8% vs. 66.7%; nausea: 27.8% vs. 44.4%; drowsiness: 44.4% vs. 55.6%; confusion: 16.7% vs. 27.8%; itching: 8.3% vs. 19.4%.

**Conclusions:**

Both strategies effectively controlled neuropathic pain, but according to the adopted selection design arm A is preferable to arm B for pain control.

**Trial Registration:**

ClinicalTrials.gov NCT00637975

## Introduction

Neuropathic pain is a common symptom in patients with cancer. Among patients with oncological pain, at least 1/3 is diagnosed with neuropathic pain [1]. In this setting, pain can be caused by a tumour compressing a nerve or it may be a side effect of chemotherapy and radiotherapy. The mainstay of treatment is opioid administration; however, a monotherapy is often insufficient to control neuropathic pain. It is widely recognised that some cancer pain syndromes are only partially responsive to opioids. This has led to the search for new strategies of treatment [2], [3]. Currently, adjuvant drugs, like antidepressants or anticonvulsants, are often employed in combination with the primary therapy [4].

Previously, a meta-analysis on the role of opioids in treating benign neuropathic pain showed that some opioids, particularly oxycodone, are more effective than other agents [2], [5]. In a meta-analysis by Finnerup et al. [6], anticonvulsants, particularly gabapentin, showed a favourable trade-off between harm and benefit; thus, these were considered the best candidates for combining with opioid treatments. Moreover, previous studies on the use of combination therapies in patients with cancer and neuropathic pain showed that gabapentin enhanced analgesia [7]. Pregabalin, a new anticonvulsant, appeared to be effective for relieving neuropathic pain, and it acted synergistically with oxycodone, with no additional toxicity [3]. Furthermore, pregabalin was active in patients with a resistance to gabapentin. A recent pharmaeconomics analysis demonstrated that pregabalin was cost-effective for patients with refractory neuropathic pain [8].

Gatti et al. demonstrated the effectiveness and tolerability of pregabalin combined with oxycodone in treating non-cancer pain in a large cohort of patients [9]. This combination seemed to be effective, particularly in an escalating dose strategy [10]. However, the strategy for increasing the dose of opioids or anticonvulsants is entirely empirical; currently, there are no data available to advocate any particular strategy.

Previous studies have demonstrated that genetic variations in pain-related receptors, transporters, and metabolising enzymes were related to opioid efficacy. The most interesting genes discovered were the mu and kappa opioid receptors [11]–[13]. Those studies generated intense interest in whether genetic analyses can be used to guide the choice of opioid treatment. However, as reviewed by Hirschhorn et al. [14], most studies that found candidate single nucleotide polymorphisms (SNPs) associated with outcomes could not be replicated [15], [16].

To our knowledge, no previous studies have investigated different dose escalation strategies in a combination therapy for neuropathic pain. In current clinical practice, this decision is typically made empirically by individual physicians, based on personal experience.

The present prospective study aimed to evaluate two different dose escalation strategies for combining pregabalin with oxycodone in treating patients with neuropathic pain caused by neoplasms. We also examined genetic SNPs as a potential basis for differences in drug responses.

## Methods

The protocol for this trial and supporting CONSORT checklist are available as supporting information; see Protocol S1 and Checklist S1.

This research has been approved by the Ethics Committee of Fatebenefratelli and Oftalmico Hospital in Milan and has been conducted according to the Declaration of Helsinki Principles. Five Italian institutions participated in the trial from September 2007 to December 2010. The protocol was approved by the ethics committees of each participating centre and written informed consent was obtained from all participants. The study is registered at Clinicaltrial.gov (NCT00637975, Supplementary File Neuropain Protocol).

### Patient Characteristics

Patients with cancer pain were enrolled when they had a clinical diagnosis of cancer and pain with a neuropathic component. Neuropathic pain was identified by the physician as burning pain, shooting or lancinating pain episodes, dysesthesia, or allodynia.

### Inclusion and Exclusion Criteria

The inclusion criteria were at least 18 years of age; pain that occurred within the 24-h period preceding the screening visit and was rated ≥4 on a NRS (range, 0–10) [17]; pain with a neuropathic component caused by malignant infiltration or compression of nervous structures; Performance Status score <3, according to the toxicity and response criteria of the Eastern cooperative oncology group (ECOG); and written informed consent. Exclusion criteria were serum creatinine >2 mg/ml or creatinine clearance <40 ml/min; previous or current pregabalin use; mild or severe hepatic insufficiency; iatrogenic neuropathy caused by chemotherapeutic agents; previous allergic reactions to oxycodone or pregabalin; and pregnancy or breastfeeding.

Chemotherapy crossover was not allowed in the 3 weeks before screening or throughout the study. Radiotherapy was not allowed on the lesion that caused the neuropathic pain. Hormone therapy that had been started before trial entry could be continued, but the dose could not be changed during the study.

### Study Design

**Figure 1:**
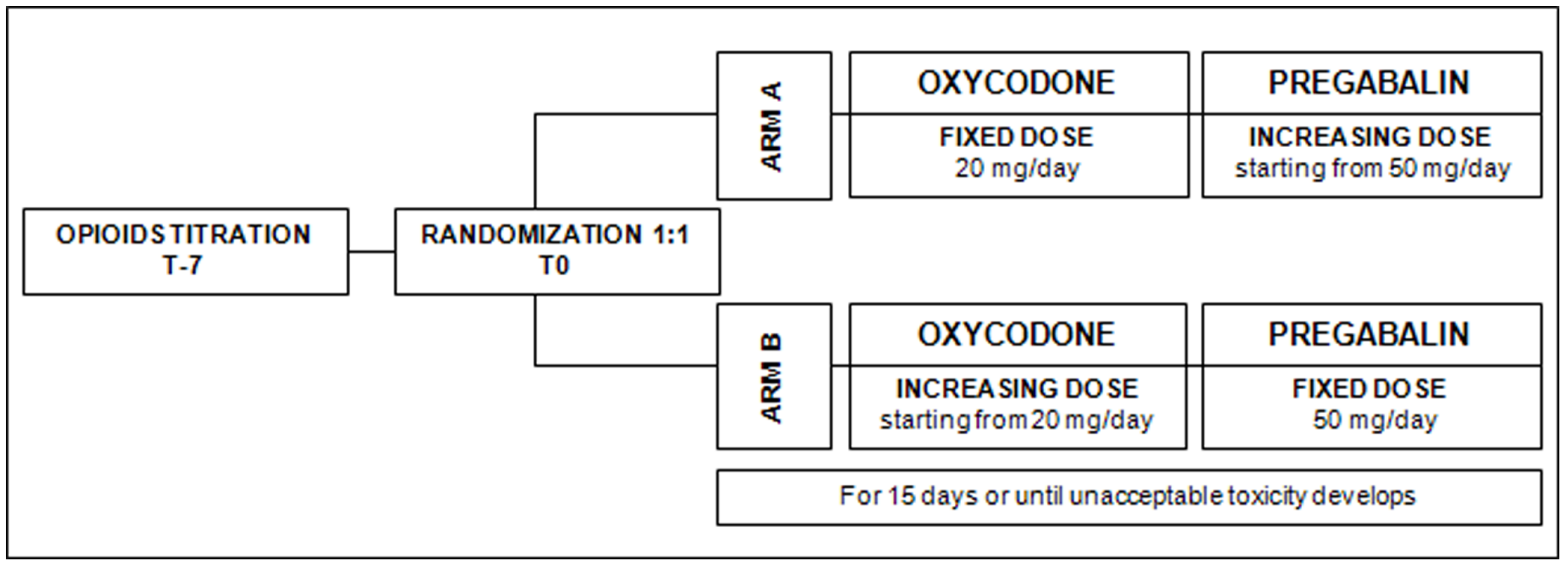
Study design.

Patients were randomly assigned by a computer-generated scheme to receive one of two dose escalation strategies. (Arm A) Patients received oxycodone controlled release (CR), 20 mg/day, plus increasing doses of pregabalin, starting at 50 mg/day. (Arm B) Patients received pregabalin, 50 mg/day, plus increasing doses of oxycodone, starting at 20 mg/day (Figure 1).

On the day of the screening visit, seven days before treatment start (−7 days), a titration phase began with the administration of normal-release morphine. On the day that treatment started (day 0), eligible patients were randomly allocated to Arm A or Arm B. After randomisation, patients were observed for 14 days. Pain and tolerability were recorded daily in a patient diary and evaluated by a physician at 3, 7, 10, and 14 days.

When the prior 24-hour pain scores, taken at specified time points, were ≥4 and no side effects were reported, the patients received a dose escalation, according to the randomisation arm. In Arm A, the dose of pregabalin was increased by 50 mg up to a maximum dose of 300 mg. In Arm B, oxycodone CR was increased in increments less than fifty percent of the previous dose. Rescue doses with normal release morphine were permitted, but they had to be recorded in the patient’s daily diary.

### Pain Assessment at Baseline

During the 24-hour period preceding the randomisation visit, average pain intensity was assessed on a 0 to 10 NRS [17], where 0 corresponded to “no pain” and 10 to “the worst possible pain”. The neuropathic component was measured with the SDN version validated in Italian [18], and “well-being” was rated with the ESAS [19].

The somatic component, mainly due to bone metastases, was also clinically evaluated by the physician and recorded on the clinical chart.

Allodynia, defined as pain in response to non-painful stimulation, was assessed by asking the patient to describe the location of a referred pain, and gently stroking the area with a cotton swab. The pain was recorded as present or absent.

### Pain Intensity Follow-Up and Pain Diary

At the screening visit (day -7), patients were instructed on how to complete a daily pain diary, which included the average intensity of global pain, evaluated with the NRS, and any concomitant medications. The patient was also required to record the number of daily breakthrough pain episodes (BTP) and the use of rescue analgesic doses. At each follow-up visit (days 3, 7, 10, and 14), pain was re-assessed by the physician with the SDNs, and ESAS interviews were performed.

### Efficacy and Safety Outcomes

The primary endpoint was overall analgesia, defined as a reduction of pain intensity by at least 1/3 (NRS). Secondary endpoints were reductions in BTP episodes and neuropathic pain (SDN) and an improvement in patient well-being (ESAS).

Drug safety was assessed by evaluating the type, frequency, and intensity of any reported adverse events according to the Common Terminology Criteria for Adverse Events Version 4.02 (CTCAE 4.02) [20].

### Ancillary Study

Within the main study, there was an optional ancillary study aimed to detect a possible correlation between selected genes and the drug response. A subset of 50 out of 76 patients from five Italian institutions participated in the ancillary study, from September 2007 to December 2010. The candidate genes included, in addition to others, the opioid receptors, kappa 1 (*OPRK1*) and mu 1 (*OPRM1*) [11]–[13]. DNA was extracted from blood samples with a Maxwell 16 DNA Purification Kit (Promega, Milan, Italy). Three *OPRK1* polymorphisms (rs7815824, rs702764, and rs1051660) and one *OPRM1* polymorphism (rs1799971) were genotyped with TaqMan SNP Genotyping assays (Applied Biosystems, Monza, Milan). PCR was carried out in 384-well plates, prepared with an automatic liquid handling system (epMotion 5075, Eppendorf, Milan Italy). The PCR-amplified DNA fragments were analysed with Allelic Discrimination Sequence Detection Software (Applied Biosystems, Monza, Italy).

### Statistical Methods

#### Design and sample size evaluation

The study adopted a randomised selection design described by Simon et al [21]. The minimum expected response rate was set to 45%, and a minimum of 74 patients was required for a power of 90% probability of correctly detecting the best schedule for a true response difference of at least of 15%. With this sample size, the study also had a ≥90% power to make the correct choice of the best treatment, even with baseline response rates greater than 45%, under the hypothesis that the true response difference was at least 15%.

#### Analysis

Analgesia, defined as at least a 1/3 reduction in pain intensity, was the primary endpoint. Analgesia was described in terms of frequency and proportion, with the relative 95% C.I. and median time-to-analgesia derived from Kaplan-Meyer curves. Changes in well-being (ESAS) and reductions in BTP episodes, allodynia, somatic pain, and side effects were reported in terms of absolute and relative frequencies or medians and ranges, according to the type of data (categorical, nominal, or continuous). Since the randomised selection design is not powered for formal comparisons between arms, the differences between the two groups were analysed only for exploratory purposes with the χ^2^ test or Wilcoxon non parametric test, according to the type of data. Significance was set at p-values<0.05.

A multivariate analysis was performed and adjusted for the presence of a somatic component.

## Results

From September 2007 to November 2010, 75 patients were randomised in 5 Italian centres. Thirty-eight (50.7%) were assigned to arm A and 37 (49.3%) were assigned to arm B.

### Study Profile

**Figure 2:**
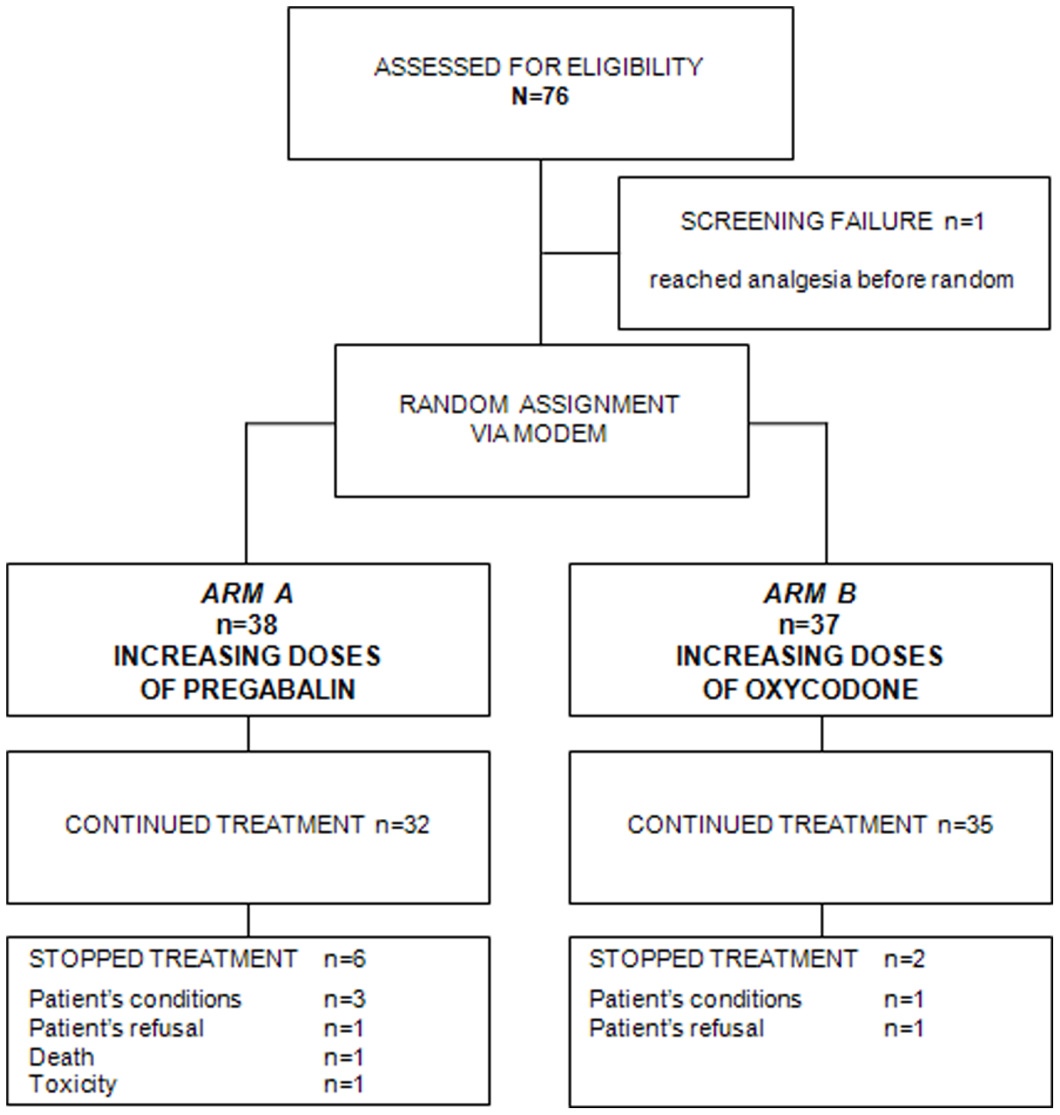
Patient CONSORT Diagram.

The trial implementation profile is shown in Figure 2 (Supplementary File Consort Checklist), according to the Consolidated Standards of Reporting Trials Statement (CONSORT). Of the 38 and 37 patients in arms A and B, 32 (84.2%) and 35 (94.6%), respectively, completed the treatment. Reasons for discontinuation included worsening of general conditions (3 patients in arm A, 1 patient in arm B), patient withdrawal (1 patient in arm A, 1 patient in arm B), death (1 patient in arm A), and adverse events (1 patient in arm A presented mental confusion, rated G3 according to the CTCAE 4.0 [20]).

### Patient Demographics and Baseline Clinical Characteristics

Demographic characteristics of patients and baseline pain characteristics are reported in Table 1. Although an imbalance was detected regarding the site of disease, the somatic component of pain was equally distributed in the two arms of treatment. All patients had received previous treatment with either weak or strong opioids.

**Table 1:** Patient demographic characteristics and baseline pain in patients with oncological neuropathic pain.

		*Arm A*		*Arm B*	
**Age (years)**	Median	68		67	
	Range	51–85		39–80	
		***N***	***(%)***	***N***	***(%)***
**Gender**	Male	21	(55.3)	17	(45.9)
	Female	17	(44.7)	20	(54.1)
**Pathology**	NSCLC	10	(26.3)	14	(37.8)
	Breast	7	(18.4)	7	(18.9)
	Colorectal	2	(5.2)	8	(21.6)
	Other	19	(50.0)	8	(21.6)
**Stage**	Non advanced	3	(7.9)	1	(2.7)
	Advanced	35	(92.1)	36	(97.3)
**Performance Status (ECOG)**	0–1	32	(84.2)	35	(94.6
	>1	6	(15.8)	2	(5.4)
**Pain (NRS scale)**	≤6	20	(52.6)	24	(64.9)
	>6	18	(47.4)	13	(35.1)
	< = 3	1	(2.6)	2	(5.4)
	4	6	(15.8)	11	(29.7)
	5	7	(18.4)	7	(18.9)
	6	6	(15.8)	4	(10.8)
	7	8	(21.1)	5	(13.5)
	8	7	(18.4)	2	(5.4)
	9	3	(7.9)	6	(16.2)

### Primary Activity Outcome

Reduction of at least 33% of the baseline pain was achieved in 26 (76.5%) patients in arm A vs. 23 (63.9%) patients in arm B (OR = 1.84; 95% CI: 0.65–5.22; p-value = 0.25). The median NRS score for pain was 6 (range 4–9) in arm A and 5 (range 2–9) in arm B. The result was confirmed in a multivariate analysis, after adjustments for gender, the presence of a somatic pain component, and the initial pain score (OR = 1.76; 95% CI: 0.61–5.05; p-value = 0.29). Analgesia was achieved in arm A with a mean dose of 100 mg of pregabalin and in arm B with a mean dose of 60 mg of oxycodone. The median time to achieve analgesia was 10 days (95% CI: 5–14 days) in arm A and 11 days (95% CI: 5–18 days) in arm B. Activity outcome is summarised in Table 2.

**Table 2:** Activity outcomes.

		*Arm A*			*Arm B*	
Pain reduction	*Patients*	*N*		*(%)*	*N*	*(%)*
	*Yes*	26		(76.5)	23	(63.9)
	*No*	8		(23.5)	13	(36.1)
	*Crude Odds Ratio (95% CI)*	1.84 (0.65–5.22)				
	*p-value*	0.25				
	*Adjusted Odds Ratio by gender and initial pain score* *(95% CI)*	1.76 (0.61–5.05)				
	*p-value*	0.29				
**Time to pain reduction**	*Median days (range)*	10 (5–14)			11 (5–18)	
	*Crude Rate of Analgesia Induction Ratio (95% CI)*	1.43 (0.76–2.70)				
	*p-value*	0.27				
	*Adjusted Rate of Analgesia Induction Ratio by gender* *and initial pain score (95% CI)*	1.41 (0.75–2.66)				
	*p-value*	0.67				
**Use of analgesic**	***Patients***	***N***	***(%)***		***N***	***(%)***
	*Yes*	10	(29.4)		8	(28.6)
	*No*	24	(70.6)		28	(71.4)
	*p-value*	0.59				

### Secondary Activity Outcomes

The frequency of the use of rescue doses of analgesics for BTP was similar between groups: arm A, 10 patients (29.4%); arm B, 8 patients (28.6%); p = 0.59.

The well-being of patients, based on the ESAS scale and SDN item distributions was not statistically different between the study arms.

### Toxicity

Unexpectedly, distinct toxicity profiles for each study arm could not be found (Table 3). In fact, there was no difference between the two study arms (constipation, drowsiness, confusion, itching, nausea).

**Table 3:** Evaluation of toxicity.

		*Arm A*		*Arm B*	
		*N*	*(%)*	*N*	*(%)*
**Constipation**	**No**	17	(47.2)	12	(33.3)
	**Yes**	19	(52.8)	24	(66.7)
		*p-value* 0.34			
**Nausea**	**No**	26	(72.2)	20	(55.6)
	**Yes**	10	(27.8)	16	(44.4)
		*p-value* 0.22			
**Drowsiness**	**No**	20	(55.6)	16	(44.4)
	**Yes**	16	(44.4)	20	(55.6)
		*p-value* 0.48			
**Confusion**	**No**	30	(83.3)	26	(72.2)
	**Yes**	6	(16.7)	10	(27.8)
		*p-value* 0.40			
**Itching**	**No**	33	(91.7)	29	(80.6)
	**Yes**	3	(8.3)	7	(19.4)
		*p-value* 0.31			
**Other**	**No**	29	(80.6)	22	(61.1)
	**Yes**	7	(19.4)	14	(38.9)
		*p-value* 0.12			

### Analysis of Polymorphisms

We analysed DNA samples from 50 patients to determine whether the *OPRK1* and *OPRM1* genes were correlated to the activity and/or toxicity of the combination of pregabalin and oxycodone. We found no significant correlations. Moreover, the distributions of the different genotypes were similar in the two groups.

## Discussion

The aim of this phase two study was to assess two different dose escalation strategies for the treatment of neuropathic pain in an oncological setting. The study was designed according to the Simon selection design in order to select the best strategy for a subsequent phase 3 trial. Regarding tolerability, we confirmed the data available in the literature, which showed that the combination of oxycodone and pregabalin was effective and safe [2], [5], [6]. In fact, adherence to the study protocol was very high: only one patient discontinued treatment based on toxicity, due to severe disorientation (G3); two other patients refused to continue treatment, one for each arm. Our results also showed that, in contrast to the daily clinical practice of many physicians, pain control can be safely achieved with incremental doses of pregabalin, and it does not require increasing doses of opioids, like oxycodone. Studies on pregabalin [22] have demonstrated that doses of up to 600 mgs daily were well tolerated. In clinical practice, sometimes the dose escalation takes place faster than recommended, and consequently, neurological toxicity occurs, like dizziness, nausea, and general malaise, which can lead to the premature interruption of treatment. Instead, we observed that, when patients gradually increased pregabalin, as in this study, they could benefit from high pregabalin doses in a safe setting.

The response to neuropathic pain treatment and the incidence of side effects are different for each patient. This variability may be explained by the specific characteristics of individual patients. Thus, we selected and evaluated potential polymorphisms in two key genes involved in the opioid response with genetic analyses of the *OPRK1* and *OPRM1* SNPs. Unfortunately, our data were not sufficiently powered to determine genetic influences on drug response. Nevertheless, the distributions of these genotypes were equivalent between the groups studied. This allowed us to exclude the possibility that the clinical differences observed between the two randomised groups were influenced by differences in mu and kappa receptor genes. Our analysis indicated that the genes identified by rs1051660, rs1799971, and rs7815624 had no interactions with the results. However, the rs702764 polymorphism showed an odds ratio of 1.8; thus, in future large scale studies, it might be interesting to analyse the role of this polymorphism in the response to combination therapy.

The treatment of neuropathic pain remains an open issue. Active research should be conducted to find more effective and personalised treatments. This paper could lead to a phase III study that would compare the strategy of oxycodone plus pregabalin at increasing doses with a conventional treatment, like gabapentin plus morphine, as published by Caraceni and colleagues [7].

## Supporting Information

Protocol S1 **Trial protocol.**
(DOC)

Checklist S1 **CONSORT checklist.**
(DOC)
