# Supplementary material for: Randomised Phase II Trial (NCT00637975) Evaluating Activity and Toxicity of Two Different Escalating Strategies for Pregabalin and Oxycodone Combination Therapy for Neuropathic Pain in Cancer Patients
Source: PLoS One. 2013 Apr 5;8(4):e59981. doi: 10.1371/journal.pone.0059981 (PMC3618180; doi:10.1371/journal.pone.0059981)
Supplement: Protocol S1 — Trial protocol. (DOC) [file pone.0059981.s001.doc]

**STUDIO DI FASE II RANDOMIZZATO**

**PER VALUTARE L’EFFICACIA E LA TOLLERABILITÀ DI OSSICODONE CR A DOSAGGIO FISSO**

**E PREGABALIN A DOSI CRESCENTI**

**VERSUS**

**OSSICODONE CR A DOSI CRESCENTI**

**E PREGABALIN A DOSAGGIO FISSO**

**NEL TRATTAMENTO DEL DOLORE NEUROPATICO IN PAZIENTI AFFETTI DA CANCRO**

**
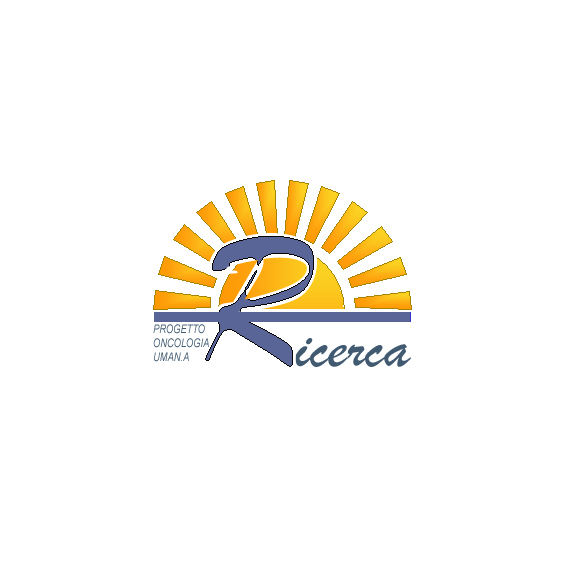
**

FASE DI SVILUPPO

Studio di fase II

CENTRI PARTECIPANTI

Ospedale Fatebenefratelli e Oftalmico, Milano

Ospedale S. Orsola, Brescia

Ospedale di Treviglio, Bergamo

Ospedali Riuniti, Bergamo

Ospedale di Legnano

Ospedale di Cremona

Istituto Nazionale Tumori, Milano

STUDIO INDIPENDENTE, NO-PROFIT

Sponsor:

Associazione Progetto Oncologia *UMAN.A*
per la ricerca (onlus)

U.O. Oncologia Medica

Ospedale Fatebenefratelli e Oftalmico, Milano

Segreteria Organizzativa:

Joanna Landi

Indice

Sinossi ………………………………………………..………………………………… 4

[Razionale ……………………………………………………………………..……. 8](#__RefHeading___Toc162923058)

[Obiettivo ……………………………………………………………………....……. 13](#__RefHeading___Toc162923059)

[Disegno …………………………………………………………………………….…. 13](#__RefHeading___Toc162923060)

[Popolazione ……………………………………………………….……………. 14](#__RefHeading___Toc162923061)

[Trattamenti ……………………………………………………………………… 15](#__RefHeading___Toc162923062)

[Considerazioni statistiche ……………………..…………… 17](#__RefHeading___Toc162923063)

[Bibliografia ………………………………………………..…………………… 19](#__RefHeading___Toc162923064)

[Allegati ………………………………………………………….………………….. 21](#__RefHeading___Toc162923065)

# SINOSSI

| Titolo dello Studio | |  |
| --- | --- | --- |
| **Studio di fase II randomizzato per valutare l’efficacia e la tollerabilità di ossicodone CR a dosaggio fisso e pregabalin a dosi crescenti versus ossicodone CR a dosi crescenti e pregabalin a dosaggio fisso nel trattamento del dolore neuropatico in pazienti affetti da cancro** | | |
| CODICE dello Studio | NEUROPAIN01 | |
| Sponsor | ASSOCIAZIONE PROGETTO ONCOLOGIA UMAN.A (onlus) | |
| Studio di fase | II | |
| Centri partecipanti allo Studio | Ospedale Fatebenefratelli e Oftalmico, Milano  Ospedale S. Orsola, Brescia  Ospedale Fatebenefratelli, Benevento  Ospedali Riuniti, Bergamo  Ospedale di Legnano  Ospedale di Cremona  Ospedale Fatebenefratelli, Roma  Ospedale Sacro Cuore, Rovigo | |
| Periodo studio | Primo paziente: giugno 2007  Durata del reclutamento: 2 annI | |
| Obiettivi Studio | **Obiettivo primario:**   - Raggiungimento dell’analgesia  (riduzione di almeno 1/3 dell’intensità del dolore)   **Obiettivi secondari:**  - Velocità nel raggiungere il controllo del dolore - Riduzione del n° dei DEI (Dolore Episodico Intenso) - Rilevazione degli effetti collaterali (ESAS) - Riduzione dell’allodinia in pazienti che la presentano al T0 - Soddisfazione del paziente - Incremento dosi di oppioidi e/o pregabalin necessarie per   controllare il dolore. - Studio ancillare da effettuarsi sul materiale ematico delle pazienti   (polimorfismi). | |
| Disegno Studio | Questo studio multicentrico randomizzato di fase II arruolerà pazienti affetti da cancro che presentano dolore con componente neuropatica.  Il dolore neuropatico verrà valutata mediante la Scala del Dolore Neuropatico (SDN) validata in Italiano.  Ai pazienti verrà richiesto di effettuare prelievi ematici a T0 sul quale verrà effettuata la valutazione dei polimorfismi.  I pazienti verranno randomizzati al braccio A o al braccio B, dopo una titolazione con ossicodone CR per tutti i pazienti in trattamento con altri oppioidi.  **Braccio A: ossicodone CR a dosaggio fisso e pregabalin a dosi crescenti.**  **Braccio B: ossicodone CR a dosi crescenti e pregabalin a dosaggio fisso.**  L’efficacia e la sicurezza di entrambi i bracci di trattamento verrà monitorata periodicamente (dopo 3, 7, 10 e 14 giorni dall’inizio della terapia) mediante la somministrazione di questionari che rilevano la risposta al trattamento, l’intensità del dolore, la rilevazione degli eventi avversi.  Il paziente verrà visitato in ambulatorio al T0, T7, T14 e verrà contattato telefonicamente al T3 e T10 | |
| Numero pazienti da arruolare | 80 | |
| Dosaggi farmaci durante lo studio | **Fase di titolazione con ossicodone CR.**  I pazienti in trattamento con ossicodone CR, da almeno 1 settimana, verranno direttamente randomizzati.  I pazienti in trattamento con oppioidi diversi da ossicodone CR, effettueranno una titolazione con ossicodone CR (dosi equianalgesiche). La durata della fase di titolazione è prevista di 7 giorni. Al termine di tale periodo, verranno randomizzati.  **Braccio A:**  **Ossicodone CR a dose fissa e pregabalin a dosi crescenti**  Ossicodone CR al dosaggio in uso da parte del singolo paziente.  Pregabalin a partire da 50 mg (25 mg 2 volte al giorno).  Se il dolore non fosse controllato si aumenterà il dosaggio di pregabalin di 50 mg ogni 3- 7 giorni, secondo la necessità e tenuto conto degli eventuali effetti collaterali, fino al dosaggio massimo di 300 mg.  E’ prevista una dose rescue in caso di DEI con morfina orale IR, il dosaggio verrà indicato dal medico curante, in considerazione della dose di oppioidi già in uso dal paziente.  **Braccio B:**  **Ossicodone CR a dosi crescenti e pregabalin a dose fissa**  Ossicodone CR al dosaggio già in uso da parte del singolo paziente.  Se il dolore non fosse controllato si aumenterà il dosaggio dell’ossicodone CR potrà essere aumentato ogni 24 ore, di non più del 50% della dose iniziale e tenuto conto degli eventuali effetti collaterali.  Pregabalin al dosaggio di 50 mg die.  E’ prevista una dose rescue in caso di DEI con morfina orale IR, il dosaggio verrà indicato dal medico curante, in considerazione della dose di oppioidi già in uso dal paziente. | |
| Criteri inclusione ed esclusione | **Criteri di inclusione:**   - Consenso informato scritto - Pazienti (sesso maschile o femminile) ≥ 18 anni - Diagnosi strumentale e clinica di neoplasia maligna - Presenza di dolore con componente neuropatica, secondo parere del medico curante - Presenza di un livello di dolore ≥ 4 (NRS) - PS ECOG <3 - Pazienti in terapia con FANS, oppioidi minori o oppioidi maggiori. - Pazienti non in terapia con Pregabalin (sono includibili i pazienti trattati con Gabapentin)   **Criteri di esclusione:**   - Creatinina serica > 2 mg/ml o creatinina clearance < 40 ml/min. - Insufficienza epatica moderata-severa - Radioterapia sulla lesione dolorosa a partire dai 15 giorni precedenti dall’entrata in studio e per tutta la durata dello studio. - Modificazioni del trattamento antitumorale (chemioterapia, ormonoterapia) per tutta la durata dello studio. - Pazienti affetti da neuropatie iatrogene da chemioterapici - Precedenti reazioni allergiche di grado 3-4 all’ossicodone e pregabalin - Gravidanza o allattamento - Insufficienza respiratoria moderata-severa - Pazienti noti per abuso di alcolici, psicofarmaci e stupefacenti ad uso diversionale. | |
| Durata programmata  del trattamento per ogni singolo paziente | La durata del trattamento è prevista di 14 giorni (+ 7 giorni se necessita la fase di titolazione con ossicodone CR).  Il trattamento terminerà se si dovessero verificare i seguenti casi:   - Tossicità inaccettabile per il paziente - Sintomatologia dolorosa non controllata dalla terapia, anche dopo i programmati aumenti di dose, o che necessiti dell’aggiunta di farmaci adiuvanti (cortisone...) non utilizzati all’ingresso nello studio - Ritiro del consenso | |
| tollerabilità | Valutazione del benessere e degli effetti collaterali (scala ESAS). | |
| Dimensione campionaria | Lo studio adotterà un disegno randomizzato di selezione, secondo l’approccio di Simon, Wittes ed Ellemberg (Randomized phase II clinical trials. Cancer treatment reports 1985; 69: 1375-1381).  In base a tale approccio verrà selezionata per la fase III la combinazione in cui si riscontra la maggior proporzione di pazienti con analgesia adeguata.  Lo studio ha una probabilità del 90% di individuare correttamente la combinazione più attiva qualora la differenza ‘vera’ tra queste sia almeno del 15%.  Si postula che la proporzione minima di analgesia sia del 45%.  Sotto queste condizioni occorreranno 37 pazienti per braccio.  Tale numero verrà portato a 40 per tenere conto di possibili drop-outs. | |
| Metodi statistici | Le caratteristiche socio-demografiche e cliniche della popolazione in studio verranno descritte per braccio di trattamento utilizzando statistiche riassuntive appropriate.  Le proporzioni di pazienti con analgesia verranno riportate con i relativi limiti di confidenza calcolati in base alla distribuzione binomiale.  Tale approccio verrà utilizzato anche per la descrizione del numero di episodi di BTP e di effetti collaterali.  Il tempo al controllo del dolore verrà descritto tramite approcci di analisi della sopravvivenza.  Il confronto tra i due gruppi, di tipo esplorativo, utilizzerà il test esatto di Fisher per le variabili categoriche ed il modello semiparametrico di Cox per l’analisi del tempo al controllo del dolore e la sua associazione con potenziali fattori prognostici.. | |
| STUDI ANCILLARI | Valutazione dei polimorfismi del gene OPRM1,del gene COMT, e del ABCB1  La valutazione necessiterà di campioni di sangue prestabiliti che verranno centralizzati all’Ospedale Fatebenefratelli e Oftalmico di Milano. | |

# Razionale

Il numero delle nuove diagnosi di neoplasia che annualmente vengono registrare sembrano essere proporzionate alla dimensione demografica di ogni nazione occidentale: ai 1.300.000 nuovi casi di tumore/anno negli USA (con circa 555.000 morti) corrispondono in Italia 300.000 nuovi casi con una mortalità stimata di 160.000 e con una prevalenza di ca. il 3%.

Il dolore moderato-severo rappresenta nella maggior parte dei pazienti oncologici uno dei sintomi più frequenti, fino all’80-90% dei pazienti con malattia metastatica o localmente avanzata. Tale sintomatologia interferisce grandemente con la qualità di vita, con il sonno e con le normali attività quotidiane.

Nonostante i recenti progressi nel trattamento farmacologico delle neoplasie ed alcuni recenti progressi nella terapia del dolore, il sintomo dolore è ancora sottotrattato; poco infine si è fatto soprattutto nel dolore con componente neuropatica, che rappresenta una delle più frequenti presentazioni del dolore in oncologia.

Il dolore neuropatico è un dolore dovuto ad una lesione primaria del sistema nervoso o derivante da una disfunzione dello stesso (1).

E’ importante riconoscere il dolore neuropatico e differenziarlo da tipi di dolore di diversa origine fisiopatologia perché il suo trattamento è diverso.

La fisiopatologia dei diversi tipi di dolore è alla base delle differenti presentazioni e delle risposte ai diversi farmaci antidolorifici.

L’attivazione dei recettori superficiali delle strutture superficiali e profonde, così come dei visceri, da parte di stimoli meccanici e chimici eccita le terminazioni afferenti della via del dolore; i messaggi non-nocicettivi sono trasmessi attraverso le fibre rapide A-β mentre le informazioni nocicettive vengono trasmesse attraverso le fibre afferenti a conduzione lenta A-δ e attraverso le fibre C.

Le terminazioni recettoriali delle fibre A-δ rispondono spesso ad un singolo stimolo, mentre la maggior parte dei recettori delle fibre C rispondono a stimoli multipli. Queste afferenze sensoriali primarie hanno il loro corpo cellulare nelle radici dei gangli dorsali. Le connessioni sinaptiche di queste afferenze primarie con il corrispondente neurone nocicettivo secondario delle corna spinali dorsali, rappresentano la sede iniziale di processazione delle informazioni sensitive e si comportano come un realis nella trasmissione dei segnali dolorosi al Sistema Nervoso Centrale (SNC). La trasmissione sensoriale è mediata dall’azione di aminoacidi eccitatori come il glutammato, l’aspartato, la sostanza P e il N-metil-D-aspartato (NMDA). Attraverso l’azione degli aminoacidi eccitatori, il flusso di calcio e la mobilizzazione del calcio intracellulare comportano la modificazione del sistema dei secondi messaggeri; una delle attivazioni del sistema dei secondi messaggeri è la formazione di ossido di azoto (NO). Si verifica inoltre un aumento nella trascrizione c-fos, che può modulare l’espressione dei geni degli oppioidi endogeni (pre-proencefalina, pre-prodinorfina). L’attivazione del recettore di NMDA promuove e mantiene l’attivazione sensitiva a livello centrale, la quale è considerata uno dei più importanti meccanismi del dolore neuropatico (2).

A livello dei neuroni secondari nelle corna dorsali, si verificano interazioni tra trasmettitori liberati dalle afferenze primarie tra cui acido amino-butirrico (GABA), glicina, adenosina, bombesina ed encefalica. Queste vie ascendenti si proiettano su regioni particolari di talamo e corteccia. Vie discendenti afferiscono alle corna dorsali per modulare la trasmissione del dolore.

I recettori degli oppioidi sono localizzati a livello delle vie ascendenti e discendenti della via di trasmissione del dolore. Questi recettori mediano i multipli effetti farmacologici degli oppioidi analgesici.

Convenzionalmente la diagnosi del dolore neuropatico viene posta dopo un attento esame del medico specialista in terapia del dolore e/o da parte del medico che ha in cura il paziente; un’altra opportunità è l’utilizzo di apposite scale di valutazione (SDN, validazione italiana del Neuropathic Pain Scale) (3).

Il dolore è un sintomo presente nel 40-90% dei pazienti affetti da cancro (3). Circa il 34% di essi sperimenta dolore neuropatico (4, 5).

| **Tipo di tumore** | **N pazienti** | **% pazienti** | **Totale** |
| --- | --- | --- | --- |
| Somatico (osso, parti molli) | 744 | 35 |  |
| Viscerale | 354 | 17 |
| Somatico e viscerale | 273 | 13 |
| Neuropatico | 185 | 9 | 34% |
| Somatico e neuropatico | 444 | 21 |
| Viscerale e neuropatico | 39 | 2 |
| Somatico, viscerale e neuropatico | 36 | 2 |
| Non noto | 43 | 2 |  |

Il dolore neuropatico viene spesso riferito come “bruciore”, “a scossa”, “tagliente”, “a fitte lancinanti”, “dolore di fondo sordo” (tipo mal di denti), “dolore al tocco o allo sfioramento” (allodinia) e si presenta spesso associato ad alcuni sintomi come parestesie, disfunzioni autonomiche (la cute della regione dolente può diventare improvvisamente rossa) e disestesie.

In alcuni gruppi di pazienti, per esempio in pazienti affetti da neoplasie della testa e collo, si verifica un’alta incidenza di dolore neuropatico (nel 25-60% dei casi) (6).

Il dolore neuropatico può essere causato dall’infiltrazione o dalla compressione di strutture nervose da parte della massa neoplastica.

Esempi di dolore neuropatico possono essere il dolore indotto dalle compressioni dei plessi, da neuropatie indotte da chemioterapici, da neuropatie paraneoplastiche o le sindromi dell’arto fantasma. Molto spesso, tuttavia, questo tipo di dolore si presenta in associazione ad altre componenti ed è caratterizzato, rispetto al dolore somatico e viscerale, da una difficile gestione farmacologica, spesso poco responsiva a varie opzioni terapeutiche. (7)

Gli oppioidi sono storicamente i farmaci usati per il trattamento del dolore da cancro moderato-forte, ma si sono dimostrati non ottimali nel controllo del dolore neuropatico eventualmente associato.

Per questo motivo si sono associati agli oppioidi, farmaci adiuvanti, di solito anticonvulsivanti e antidepressivi (8, 9) e, più recentemente, il gabapentin, appartenente alla nuova generazione degli anticonvulsivanti (10, 11, 12) per aumentarne il potere analgesico.

La morfina, oltre ad essere raccomandata dall’OMS come farmaco di riferimento per il trattamento del dolore severo, associato o non al cancro, viene anche usata nel trattamento del dolore neuropatico (13, 14).

L’ossicodone, oppioide simile alla morfina ma con maggior biodisponibilità ed emivita più breve, viene utilizzato in molti casi di dolore da cancro. Studi di confronto tra ossicodone e morfina hanno evidenziato tollerabilità ed efficacia simile per i due oppioidi, con simili effetti collaterali. E’ da segnalare la riduzione del prurito e la riduzione di allucinazioni nei pazienti trattati con ossicodone CR (15, 16). Inoltre, in uno studio recentemente pubblicato (17), si segnala che pazienti non responders alla morfina CR abbiano ottenuto un’alta percentuale di riposta se trattati con ossicodone CR.

L’ossicodone trova il suo utilizzo anche nel trattamento del dolore neuropatico
(18, 19).

Il gabapentin ha ottenuto buoni risultati nel controllo del dolore neuropatico da neuropatia diabetica e, in associazione con oppioidi, anche nel dolore neuropatico da infiltrazione tumorale (20, 21).

Il pregabalin ha un profilo farmacologico simile al gabapentin, ma ha dimostrato una più elevata attività analgesica in studi preclinici. L’esatto meccanismo d’azione del pregabalin non è stato ancora chiarito, sebbene si ritenga che possa ridurre il rilascio di neurotrasmettitori eccitatori attraverso il legame con la subunità proteica α2δ dei canali del calcio voltaggio dipendenti. (22)

Pregabalin ha ottenuto buoni risultati nel trattamento del dolore neuropatico da neuropatia diabetica e nella nevralgia post-erpetica (23, 24).

Un recente studio, ancora in press, su 169 pazienti trattati per dolore neuropatico non correlato al cancro, trattato con ossicodone CR e pregabalin, ha evidenziato una riduzione importante di tale dolore (NRS al T0 7,43 e NRS T a 85 giorni di 1,49) (25).

In considerazione dei dati presenti in letteratura, da cui risulta l’attività dell’oppioide ossicodone e degli anticonvulsivanti gabapentin e pregabalin nel trattamento del dolore neuropatico, della fisiopatologia della via di trasmissione del dolore e della soddisfacente tollerabilità di gabapentin associato all’oppioide ossicodone si è deciso di proporre uno studio che preveda l’utilizzo di ossicodone e di pregabalin.

La scelta di associare nel nostro studio pregabalin all’oppioide ossicodone CR, utilizzato ormai stabilmente e con soddisfacenti risultati in pazienti che presentano dolore neuropatico cancro-correlato, può, a nostro avviso, essere una combinazione con buone possibilità di risposta.

Lo studio prevede dosaggi crescenti dei due farmaci e ciò permetterà di monitorare attentamente il profilo tossicologico dell’associazione e dei singoli farmaci.

La scelta di randomizzare in 2 gruppi i pazienti, con, alternativamente, un farmaco a dosaggio fisso ed il secondo, con dosaggi crescenti al bisogno, è volta ad individuare la migliore strategia per il trattamento di questo particolare tipo di dolore.

Si considera importante tale associazione in un campo, come quello del dolore neuropatico associato al cancro, in cui non esistono definite linee guida. per la risoluzione della sintomatologia.

## Studi ancillari

Uno dei campi più esplorati recentemente nella terapia del dolore è quello che tenta di valutare l’eventuale presenza di polimorfismi genici in associazione alla risposta terapeutica agli oppiacei.

Attualmente i geni più esplorati sono quelli che codificano per il recettore
μ OPRM1 e quello che codifica per la catecolmetiltranferasi COMT.

Questi geni sono stati ampiamente studiati nell’associazione alla risposta alla morfina, dove si è trovata una forte associazione tra associazione e risposta (26). Questa associazione non è mai stata studiata con l’ossicodone.

Scopo del nostro studio ancillare è pertanto valutare se esitano polimorfismi associati ad una maggiore efficacia dei farmaci in uso.

# Obiettivo

Valutazione dell’efficacia e della tossicità dell’associazione di ossicodone a rilascio controllato in associazione a pregabalin in due strategie terapeutiche differenti

## Endpoint primario

- Raggiungimento dell’analgesia (riduzione di almeno 1/3 dell’intensità del dolore)
   valutata mediante la scala NRS.

## Endpoint secondari

- Velocità nel raggiungere il controllo del dolore
- Riduzione del n° dei DEI (Dolore Episodico Intenso)
- Rilevazione degli effetti collaterali durante il trattamento
- Riduzione della allodinia per i pazienti che la presentano al T0
- Studio di farmacocinetica, farmacodinamica e ricerca dei polimorfismi, a tempi
   prestabiliti.
- Valutazione del raggiungimento del benessere (scala ESAS).
- Raggiungimento dell’analgesia (scala SDN)

# Disegno

Studio multicentrico randomizzato su pazienti affetti da neoplasia che presentino dolore neuropatico, valutato mediante le Scale (NRS, SDN).

I pazienti verranno randomizzati al braccio A o al braccio B, dopo una titolazione con ossicodone per tutti i pazienti in trattamento con altri oppioidi.

Tale fase durerà 7 giorni.

**Braccio A:** *ossicodone* CR a dosaggio *fisso* e pregabalin a dose crescenti.

**Braccio B**: ossicodone CR a dosi crescenti e *pregabalin* a dosaggio *fisso*.

Verrà valutato inoltre il raggiungimento del benessere e del sollievo mediante la
 scala ESAS.

L’efficacia e la sicurezza di entrambi i bracci verrà monitorata nel tempo.

# Popolazione

Pazienti affetti da neoplasia che presentano dolore neuropatico, valutato mediante la scala numerica (NRS) e la Scala del Dolore Neuropatico (SDN). L’intensità del dolore deve essere ≥ 4 (NRS). I pazienti possono essere in terapia con FANS, oppioidi minori o maggiori.

Il numero dei pazienti da arruolare, supportato da considerazioni statistiche, è di 80 (40 per ogni gruppo di trattamento).

## Criteri di inclusione

- Consenso informato scritto
- Pazienti (sesso maschile o femminile) ≥ 18 anni
- Diagnosi strumentale e clinica di neoplasia maligna
- Presenza di dolore con componente neuropatica, secondo parere del
   medico curante
- Presenza di un livello di dolore ≥ 4 (NRS)
- PS ECOG <3
- Pazienti in terapia con FANS, oppioidi minori o oppioidi maggiori
- Pazienti non in terapia con Pregabalin (sono includibili i pazienti trattati con Gabapentin).

***Criteri di esclusione***

- Creatinina serica > 2 mg/ml o creatinina clearance < 40 ml/min.
- Insufficienza epatica moderata-severa
- Radioterapia sulla lesione dolorosa a partire dai 15 giorni precedenti
   dall’entrata in studio e per tutta la durata dello studio.
- Modificazioni del trattamento antitumorale (chemioterapia, ormonoterapia)
   per tutta la durata dello studio.
- Pazienti affetti da neuropatie iatrogene ad chemioterapici
- Precedenti reazioni allergiche di grado 3-4 all’ossicodone e pregabalin
- Gravidanza o allattamento
- Insufficienza respiratoria moderata-severa
- Pazienti noti per abuso di alcolici, psicofarmaci e stupefacenti ad uso
   diversionale.

# Trattamenti

I pazienti saranno assegnati in modo randomizzato a ricevere:

**Braccio A:**

**Ossicodone CR a dose fissa e pregabalin a dosi crescenti**

Ossicodone CR al dosaggio in uso da parte del singolo paziente.

Pregabalin a partire da 50 mg (25 mg 2 volte al giorno), o dosaggio dello stesso al momento dell’ingresso nello studio.

Se il dolore non fosse controllato si aumenterà il dosaggio di pregabalin di 50 mg ogni 3-7 giorni, secondo la necessità e tenuto conto degli eventuali effetti collaterali, fino al dosaggio massimo di 300 mg.

E’ prevista una dose rescue in caso di DEI con morfina orale IR, il dosaggio verrà indicato dal medico curante, in considerazione della dose di oppioidi già in uso dal paziente.

**Braccio B:**

**Ossicodone CR a dosi crescenti e pregabalin a dose fissa**

Ossicodone CR al dosaggio già in uso da parte del singolo paziente.

Se il dolore non fosse controllato si aumenterà il dosaggio dell’ossicodone CR potrà essere aumentato ogni 24 ore, di non più del 50% della dose iniziale, e tenuto conto degli eventuali effetti collaterali.

Pregabalin al dosaggio di 50 mg die.

E’ prevista una dose rescue in caso di DEI con morfina orale IR, il dosaggio verrà indicato dal medico curante, in considerazione della dose di oppioidi già in uso dal paziente.

Il trattamento in studio può essere interrotto in seguito ad una delle seguenti circostanze:

- Eventi avversi
- Valori di laboratorio anormali clinicamente rilevanti
- Risultati strumentali anormali clinicamente rilevanti
- Violazione del protocollo
- Perdita al follow-up
- Tossicità inaccettabile per il paziente
- Sintomatologia dolorosa non controllata dalla terapia, anche dopo i programmati
   aumenti di dose, o che necessiti dell’aggiunta di farmaci adiuvanti (cortisone...)
   non utilizzati all’ingresso nello studio
- Ritiro del consenso

## Schema delle visite e delle valutazioni

|  | **Visita inclusione  e random  (T-7)** | **Visita di randomizzazione (T0)** | **T3** | **T7** | **T10** | **T14** |
| --- | --- | --- | --- | --- | --- | --- |
| **DATI DEMOGRAFICI E CLINICI** | **🗸** |  |  |  |  |  |
| **VALUTAZIONE DOLORE NEUROPATICO (SDN)** | **🗸** | **🗸** |  | **🗸** |  | **🗸** |
| **VALUTAZIONE DEL DOLORE (NRS)*** | **🗸** | **🗸** | **🗸** | **🗸** | **🗸** | **🗸** |
| **VALUTAZIONE DEL BENESSERE (ESAS)** |  | **🗸** |  | **🗸** |  | **🗸** |
| **CONTATTI TELEFONICI** |  |  | **🗸** |  | **🗸** |  |
| **CONSENSO INFORMATO** | **🗸** |  |  |  |  |  |
| **CRITERI INCLUSIONE E ESCLUSIONE** | **🗸** |  |  |  |  |  |
| **TEST GRAVIDANZA** | **🗸** |  |  |  |  |  |
| **E.O.** | **🗸** | **🗸** |  | **🗸** |  | **🗸** |
| **PS** | **🗸** | **🗸** |  | **🗸** |  | **🗸** |
| **RANDOMIZZAZIONE** |  | **🗸** |  |  |  |  |
| **VALUTAZIONE ALLODINIA  (se presente)** | **🗸** | **🗸** |  | **🗸** |  | **🗸** |
| **ESAMI EMATOCHIMICI** |  | **🗸** |  |  |  | **🗸** |
| **DIARIO** | **🗸🗸🗸🗸🗸🗸🗸** | **🗸🗸🗸** | **🗸🗸🗸🗸** | **🗸🗸🗸** | **🗸🗸🗸🗸** |  |
| **PRELIEVI PER POLIMORFISMI** |  | **🗸** |  |  |  |  |

* il dolore che il paziente riferirà è da considerarsi “puntuale”, cioè quello che presenta alla visita.

Il paziente dovrà compilare un *diario giornaliero* in cui dovrà segnalare i seguenti dati:

- valutazione del dolore (scala NRS)
- episodi di Dolore Episodico Intenso (DEI), ed analgesici eventualmente assunti al bisogno per quell’episodio (sono consentiti nello studio l’utilizzo di FANS e morfina IR)
- momento in cui (nella fase di titolazione) aumenta la dose del farmaco (a seconda del braccio, ossicodone o pregabalin)
- Il questionario Edmonton Symptom Assessment Scale (ESAS), valuta la percezione della qualità di vita che ha il paziente. (Almeno 3 volte in tutta la durata dello studio, T0,T7,T14)

La valutazione della tossicità verrà effettuata mediante la rilevazione degli effetti collaterali correlati e anche mediante esami ematochimici di funzionalità epatica, renale ed emocromo eseguiti al tempo T-7 o T0, T14.

# Considerazioni statistiche

## Disegno e dimensione campionaria

Lo studio adotterà un disegno randomizzato di selezione, secondo l’approccio di Simon, Wittes ed Ellemberg (27). In base a tale approccio verrà selezionata per la fase III la combinazione in cui si riscontra la maggior proporzione di pazienti con analgesia adeguata.

Lo studio ha una probabilità del 90% di individuare correttamente la combinazione più attiva qualora la differenza ‘vera’ tra queste sia almeno del 15%. Si postula che la proporzione minima di analgesia sia del 45%. Sotto queste condizioni occorreranno 37 pazienti per braccio. Tale numero verrà portato a 40 per tenere conto di possibili drop-outs.

La randomizzazione sarà centralizzata ed avverrà attraverso un sistema remoto, con collegamento via web.

Si prevede una stratificazione per centro con blocchi di permutazione a dimensione variabile.

## Analisi

Le caratteristiche socio-demografiche e cliniche della popolazione in studio verranno descritte per braccio di trattamento utilizzando statistiche riassuntive appropriate. Le proporzioni di pazienti con analgesia verranno riportate con i relativi limiti di confidenza calcolati in base alla distribuzione binomiale.

Tale approccio verrà utilizzato anche per la descrizione del numero di episodi di BTP e di effetti collaterali.

Il tempo al controllo del dolore verrà descritto tramite approcci di analisi della sopravvivenza.

Il confronto tra i due gruppi, di tipo esplorativo, utilizzerà il test esatto di Fisher per le variabili categoriche ed il modello semiparametrico di Cox per l’analisi del tempo al controllo del dolore e la sua associazione con potenziali fattori prognostici.

## Studi ancillari

Al tempo T-7 o T0 verrà eseguito un prelievo per l’analisi dei polimorfismi genici (geni che codificano per il recettore μ OPRM1 e per la catecolmetiltranferasi COMT, geni del metabolismo e in particolare i geni della famiglia dei citocromi P450 (CYP3A4, CYP2B etc) che possono alterare la farmacocinetica e farmacodinamica dei farmaci o trasportatori che modificano il contenuto intracellulare dei farmaci come ABCB1).

I prelievi verranno centralizzati presso l’Istituto Mario Negri, Laboratorio di Genetica Molecolare.

Le procedure sono descritte negli allegati.

# Bibliografia

1. Merskey H, Bugduk N. *Classification of chronic pain. Description of
   chronic pain. Description of chronic pain syndromes and definitions of chronic pain terms.* Seattle: IASP, press 1994
2. Elliott KG. *Taxonomy and mechanisms of neuropathic pain*. Semin Neurol 1994; 14: 195
3. Negri E, Bettaglio R, Demartini L, Allegri M, Barbieri M, Miotti D, Paulin L, Buonocore M, Bonezzi C. Related Articles, Links [Validation of the Italian version of the "Neuropathic Pain Scale" and its clinical applications] Minerva Anestesiol. 2002 Mar;68(3):95-104. Italian.
4. Caraceni A. *Clinicopathologic correlates of common cancer pain syndromes*. Hematol Oncol Clin North Am. 1996 Feb;10(1):57-78.
5. Zech DF, Grond S, Lynch J, Hertel D, Lehmann KA. *Validation of World Health Organization Guidelines for cancer pain relief: a 10-year prospective study. Pain.* 1995 Oct;63(1):65-76.
6. Chua KS, Reddy SK, Lee MC, Patt RB. *Pain and loss of function in head and neck cancer survivors*. J Pain Symptom Manage. 1999 Sep;18(3):193-202.
7. Portenoy RK, Foley KM, Inturrisi CE. *The nature of opioid responsiveness and its implications for neuropathic pain: new hypotheses derived from studies of opioid infusions.* Pain. 1990 Dec;43(3):273-86.
8. Grond S, Zech D, Diefenbach C, Radbruch L, Lehmann KA. *Assessment of cancer pain: a prospective evaluation in 2266 cancer patients referred to a pain service.* Pain. 1996 Jan;64(1):107-14.
9. Mercadante S, Arcuri E, Tirelli W, Villari P, Casuccio A. *Amitriptyline in neuropathic cancer pain in patients on morphine therapy: a randomized placebo-controlled, double-blind crossover study.* Tumori. 2002 May-Jun;88(3):239-42.
10. Caraceni A, Zecca E, Bonezzi C, Arcuri E, Yaya Tur R, Maltoni M, Visentin M, Gorni G, Martini C, Tirelli W, Barbieri M, De Conno F. *Gabapentin for neuropathic cancer pain: a randomized controlled trial from the Gabapentin Cancer Pain Study Group.* J Clin Oncol. 2004 Jul 15;22(14):2909-17.
11. Dirks J, Petersen KL, Rowbotham MC, Dahl JB. *Gabapentin suppresses cutaneous hyperalgesia following heat-capsaicin sensitization*. Anesthesiology. 2002 Jul;97(1):102-7.
12. Farrar JT, Portenoy RK. *Neuropathy cancer pain: the role of adjuvant analgesics.* Oncology (Willinston Park) 2001 Nov; 15(11):1435-42,1445; discussion 1445,1450-3.
13. Gralow I. *Cancer pain: an update of pharmacological approaches in pain therapy.* Curr Opin Anaesthesiol. 2002 Oct;15(5):555-61
14. Altier N, Dion D, Boulanger A, Choiniere M. *Management of chronic neuropathic pain with methadone: a review of 13 cases*. Clin J Pain. 2005 Jul-Aug;21(4):364-9.
15. Bruera E, Belzile M, Pituskin E, Fainsinger R, Darke A, Harsanyi Z, Babul N, Ford I. *Randomized, double-blind, cross-over trial comparing safety and efficacy of oral controlled-release oxycodone with controlled-release morphine in patients with cancer pain*. J Clin Oncol. 1998 Oct;16(10):3222-9
16. Mucci-Lo Russo P, Berman BS, Silberstein PT, Citron ML, Bressler L, Weinstein SM, Kaiko RF, Buckley BJ, Reder RF. *Controlled-release oxycodone compared with controlled-release morphine in the treatment of cancer pain: a randomized, double-blind, parallel-group study.* Eur J Pain. 1998;2(3):239-49.
17. Riley J, Ross JR, Rutter D, Wells AU, Goller K, du Bois R, Welsh K. *No pain relief from morphine? Individual variation in sensitivity to morphine and the need to switch to an alternative opioid in cancer patients.* Support Care Cancer 2006 Jan;14(1):56-64.
18. Kalso E. Oxycodone. J *Pain Symptom Manage*. 2005 May;29(5 Suppl): S47-56.
19. Attal N, Cruccu G, Haanpaa M, Hansson P, Jensen TS, Nurmikko T, Sampaio C, Sindrup S, Wiffen P; EFNS Task Force. *EFNS guidelines on pharmacological treatment of neuropathic pain.* Eur J Neurol. 2006 Nov;13(11):1153-69.
20. Bennett MI, Simpson KH. *Gabapentin in the treatment of neuropathic pain.* Palliat Med. 2004 Jan;18(1):5-11.
21. Ross JR, Goller K, Hardy J, Broadley K, A’hern R, William J. *Gabapentine is effective in the treatment of cancer-related neuropathic pain: a prospective, open label study*. J Palliat Med, 2005 Dec;8(6):1118-26.
22. Frampton JE, Foster RH. *Pregabalin: in the treatment of postherpetic neuralgia.* Drugs 2005;65(1):111-8;discussion 119-20.
23. Davis MP. *What is new in neuropathic pain?* Support Care Cancer. 2006 Nov 28; [Epub ahead of print]
24. Sonnet TE, Setter SM, Campbell RK. *Pregabalin for the treatment of painful neuropathy.* Expert Rev Neurother, 2006 Nov;6(11):1629-35.
25. Sabato A.F., Gatti A., IN PRESS, 2007
26. Reyes-Gibby CC, Shete S, Rakvag T, Bhat SV, Skorpen F, Bruera E, Kaasa S, Klepstad P. *Exploring joint effects of genes and the clinical efficacy of morphine for cancer pain: OPRM1 and COMT gene*. Pain. 2006 Dec 5
27. Simon R Wittes RE Ellemberg SS. *Randomized phase II clinical trials.* Cancer treatment reports 1985; 69: 1375-1381.
28. Bruera E, Kuehn N, Miller MJ, Selmser P, Macmillan K. *Links The Edmonton Symptom Assessment System (ESAS): a simple method for the assessment of palliative care patients.* J Palliat Care. 1991 Summer;7(2):6-9.
29. Caraceni A, Mendoza TR, Mencaglia E, Baratella C, Edwards K, Forjaz MJ, Martini C, Serlin RC, de Conno F, *A validation study of an Italian version of the Brief Pain Inventory (Breve Questionario per la Valutazione del Dolore).*Cleeland CS.Pain. 1996 Apr;65(1):87-92. Links

**ALLEGATI PROTOCOLLO**

1. Dichiarazione Good Clinical Practice
2. Scheda Lyrica
3. Scheda Oxycontin
4. Attestazioni di sicurezza dei componenti
5. Informazioni per il paziente
6. Consenso informato per la partecipazione allo studio
7. Consenso informato per lo studio sui fattori biologici
8. Lettera al medico di base

Allegato Protocollo 1

Dichiarazione di Good Clinical Practice

SI DICHIARA CHE

Le procedure riportate nel protocollo *“Studio di fase II randomizzato per valutare l’efficacia e la tollerabilità di ossicodone CR a dosaggio fisso e pregabalin a dose crescenti versus ossicodone CR a dosi crescenti e pregabalin a dosaggio fisso nel trattamento del dolore neuropatico in pazienti affetti da cancro”,* riguardanti la conduzione, lo svolgimento e la documentazione di questo studio sono state approntate per assicurare che si tenga fede ai principi etici riportati nella Dichiarazione di Helsinki e sue revisioni. Lo studio verrà condotto tenendo conto dei requisiti regolatori e degli adempimenti di legge. In particolare, il riferimento normativo è rappresentato dal DM 17/12/2005 sugli studi no profit.

………………….., ………………………….

In fede

___________________________________

Allegato Protocollo 2

Descrizione delle caratteristiche di Lyrica®

**RIASSUNTO DELLE CARATTERISTICHE DEL PRODOTTO**

**1. DENOMINAZIONE DEL MEDICINALE**

LYRICA 25 mg capsule rigide

LYRICA 50 mg capsule rigide

LYRICA 75 mg capsule rigide

LYRICA 100 mg capsule rigide

LYRICA 150 mg capsule rigide

LYRICA 200 mg capsule rigide

LYRICA 225 mg capsule rigide

LYRICA 300 mg capsule rigide

**2. COMPOSIZIONE QUALITATIVA E QUANTITATIVA**

LYRICA 25 mg capsule rigide: ogni capsula rigida contiene 25 mg di pregabalin.

LYRICA 50 mg capsule rigide: ogni capsula rigida contiene 50 mg di pregabalin.

LYRICA 75 mg capsule rigide: ogni capsula rigida contiene 75 mg di pregabalin.

LYRICA 100 mg capsule rigide: ogni capsula rigida contiene 100 mg di pregabalin.

LYRICA 150 mg capsule rigide: ogni capsula rigida contiene 150 mg di pregabalin.

LYRICA 200 mg capsule rigide: ogni capsula rigida contiene 200 mg di pregabalin.

LYRICA 225 mg capsule rigide: ogni capsula rigida contiene 225 mg di pregabalin.

LYRICA 300 mg capsule rigide: ogni capsula rigida contiene 300 mg di pregabalin.

Le capsule di Lyrica contengono anche lattosio monoidrato.

Per l’elenco completo degli eccipienti, vedere paragrafo 6.1.

**3. FORMA FARMACEUTICA**

- Capsula rigida.
- Capsula da 25 mg: Capsula contrassegnata con inchiostro nero dalla scritta “Pfizer” sul cappuccio e “PGN 25” sul corpo.
- Capsula da 50 mg: Capsula contrassegnata con inchiostro nero dalla scritta “Pfizer” sul cappuccio e “PGN 50” sul corpo. Il corpo è inoltre contrassegnato da una banda nera.
- Capsula da 75 mg: Capsula contrassegnata con inchiostro nero dalla scritta “Pfizer” sul cappuccio e “PGN 75” sul corpo.
- Capsula da 100 mg: Capsula contrassegnata con inchiostro nero dalla scritta “Pfizer” sul cappuccio e “PGN 100” sul corpo.
- Capsula da 150 mg: Capsula contrassegnata con inchiostro nero dalla scritta “Pfizer” sul cappuccio e “PGN 150” sul corpo.
- Capsula da 200 mg: Capsula contrassegnata con inchiostro nero dalla scritta “Pfizer” sul cappuccio e “PGN 200” sul corpo.
- Capsula da 225 mg: Capsula contrassegnata con inchiostro nero dalla scritta “Pfizer” sul cappuccio e “PGN 225” sul corpo.
- Capsula da 300 mg: Capsula contrassegnata con inchiostro nero dalla scritta “Pfizer” sul cappuccio e “PGN 300” sul corpo.

**4. INFORMAZIONI CLINICHE**

**4.1 Indicazioni terapeutiche**

Dolore neuropatico

Lyrica è indicato per il trattamento del dolore neuropatico periferico e centrale negli adulti.

Epilessia

Lyrica è indicato come terapia aggiuntiva negli adulti con attacchi epilettici parziali in presenza o in assenza di generalizzazione secondaria.

Disturbo d’Ansia Generalizzata

Lyrica è indicato per il trattamento del Disturbo d’Ansia Generalizzata (GAD) negli adulti.

**4.2 Posologia e modo di somministrazione**

Il dosaggio varia da 150 a 600 mg al giorno, suddiviso in due o tre somministrazioni.

Lyrica può essere assunto con o senza cibo.

Dolore neuropatico

Il trattamento con pregabalin può essere iniziato al dosaggio di 150 mg al giorno. In base alla risposta individuale ed alla tollerabilità del paziente, il dosaggio può essere aumentato a 300 mg al giorno dopo un intervallo da 3 a 7 giorni e, se necessario, può essere aumentato ad un dosaggio massimo di 600 mg al giorno dopo un ulteriore intervallo di 7 giorni.

Epilessia

Il trattamento con pregabalin può essere iniziato al dosaggio di 150 mg al giorno. In base alla risposta individuale ed alla tollerabilità del paziente, dopo 1 settimana il dosaggio può essere aumentato a 300 mg al giorno. Il dosaggio massimo di 600 mg al giorno può essere raggiunto dopo un’ulteriore settimana

Disturbo d’Ansia Generalizzata

Il dosaggio è 150-600 mg al giorno da somministrare in due o tre somministrazioni. La necessità del trattamento deve essere rivalutata regolarmente.

Il trattamento con pregabalin può essere iniziato al dosaggio di 150 mg al giorno. In base alla risposta individuale ed alla tollerabilità del paziente, dopo 1 settimana il dosaggio può essere aumentato a 300 mg al giorno. Dopo un’ulteriore settimana il dosaggio può essere aumentato a 450 mg al giorno.

Il dosaggio massimo di 600 mg al giorno può essere raggiunto dopo un’ulteriore settimana.

Sospensione del trattamento con pregabalin

In accordo all’attuale pratica clinica, se il trattamento con pregabalin deve essere sospeso, indipendentemente dall’indicazione, si raccomanda di effettuare la sospensione del trattamento in modo graduale nell’arco di almeno 1 settimana (vedere paragrafo 4.8).

*Pazienti con compromissione renale*

Pregabalin viene eliminato dalla circolazione sistemica principalmente mediante escrezione renale sotto forma di farmaco immodificato. Poiché la clearance di pregabalin è direttamente proporzionale alla clearance della creatinina (vedere paragrafo 5.2), la riduzione del dosaggio di pregabalin in pazienti con compromissione della funzionalità renale deve essere personalizzata in base alla clearance della creatinina (CLcr), come indicato nella Tabella 1 applicando la seguente formula:

Pregabalin viene eliminato in modo efficace dal plasma mediante emodialisi (50 % del farmaco in 4 ore). Per i pazienti sottoposti ad emodialisi, il dosaggio giornaliero di pregabalin deve essere corretto in base alla funzionalità renale. In aggiunta alla dose giornaliera, un’ulteriore dose di pregabalin deve essere somministrata subito dopo ogni seduta di dialisi della durata di 4 ore (vedere Tabella 1).

Tabella 1- Aggiustamento del dosaggio di pregabalin in base alla funzionalità renale

| Clearance della creatinina (CL_cr_)  (ml/min) | Dose Totale Giornaliera  di Pregabalin* | | | | Regime Posologico |
| --- | --- | --- | --- | --- | --- |
| Dose iniziale (mg/die) | | | Dose massima (mg/die) | | |
| ≥ 60 | 150 | | 600 | | BID o TID |
| ≥30 - <60 | 75 | | 300 | | BID o TID |
| ≥15 - <30 | 25 – 50 | | 150 | | Una volta al giorno o BID |
| < 15 | 25 | | 75 | | Una volta al giorno |
| Dose supplementare a seguito di emodialisi (mg) | | | | | |
| 25 | | 100 | | Dose singola ^+^ | |

TID = Tre somministrazioni

BID = Due somministrazioni

* La dose totale giornaliera (mg/die) deve essere suddivisa come indicato dal regime posologico per ottenere la singola dose prevista in mg

^+^ La dose supplementare è una singole dose aggiuntiva

*Uso in pazienti con compromissione epatica*

Non è necessario un aggiustamento posologico in pazienti con compromissione epatica (vedere paragrafo 5.2).

*Uso nei bambini e negli adolescenti*

Non è raccomandato l’uso di Lyrica nei bambini di età inferiore a 12 anni e negli adolescenti (12-17 anni) perché i dati a disposizione sulla sicurezza e l’efficacia in questi pazienti non sono sufficienti (vedere paragrafo 5.3).

*Uso negli anziani (al di sopra di 65 anni)*

Nei pazienti anziani può essere necessaria una riduzione della dose di pregabalin a causa di una riduzione della funzionalità renale (vedere pazienti con compromissione renale).

**4.3 Controindicazioni**

Ipersensibilità al principio attivo o ad uno qualsiasi degli eccipienti.

**4.4 Avvertenze speciali e precauzioni di impiego**

In accordo all’attuale pratica clinica, in alcuni pazienti diabetici che aumentano di peso durante il trattamento con pregabalin può essere necessario modificare il dosaggio dei farmaci ipoglicemizzanti.

Il trattamento con pregabalin è stato associato a capogiri e sonnolenza che possono aumentare, nei pazienti anziani, il rischio di lesioni accidentali (cadute). Pertanto, si dovrà consigliare ai pazienti di fare attenzione fino a quando non avranno familiarizzato con i potenziali effetti di questo farmaco.

Non ci sono dati sufficienti in base ai quali, una volta ottenuto il controllo degli attacchi epilettici mediante assunzione di pregabalin in terapia aggiuntiva, si possa sospendere il trattamento concomitante con altri farmaci antiepilettici e mantenere la monoterapia con pregabalin.

In alcuni pazienti, a seguito della sospensione di trattamenti a breve e a lungo termine con pregabalin sono stati osservati sintomi da astinenza. Sono stati segnalati i seguenti eventi: insonnia, cefalea, nausea, diarrea, sindrome influenzale, nervosismo, depressione, dolore, sudorazione e capogiri. I pazienti devono essere informati di questa evenienza prima dell’inizio del trattamento.

Per quanto concerne la sospensione del trattamento a lungo termine con pregabalin, non vi sono dati sull’incidenza e sulla gravità dei sintomi da sospensione in relazione alla durata del trattamento ed al dosaggio di pregabalin.

Durante la fase di commercializzazione del prodotto sono stati segnalati casi di scompenso cardiaco in alcuni pazienti in trattamento con pregabalin. Negli studi a breve termine, in pazienti senza cardiopatia o malattia vascolare periferica significativa non è stata osservata un’evidente associazione tra l’edema periferico e le complicanze cardiovascolari, quali ipertensione o scompenso cardiaco. Poiché i dati in pazienti con scompenso cardiaco grave sono limitati, pregabalin deve essere utilizzato con cautela in questi pazienti.

I pazienti affetti da rari problemi ereditari di intolleranza al galattosio, da deficit di Lapp lattasi, o da malassorbimento di glucosio-galattosio, non devono assumere questo medicinale.

Nel trattamento del dolore neuropatico centrale dovuto ad una lesione della colonna vertebrale l’incidenza degli eventi avversi in generale, del SNC e della sonnolenza in particolare, è aumentata. Ciò può essere attribuito ad un effetto aggiuntivo causato dai trattamenti concomitanti (p.es. agenti anti-spastici) necessari per questa patologia. Ciò deve essere tenuto in considerazione quando pregabalin viene prescritto in questa patologia.

**4.5 Interazioni con altri medicinali ed altre forme di interazione**

Poiché pregabalin viene principalmente escreto immodificato nella urine, subisce un metabolismo trascurabile nell’uomo (< 2% di una dose si ritrova nelle urine sotto forma di metaboliti), non inibisce il metabolismo dei farmaci *in vitro* e non si lega alle proteine plasmatiche, è improbabile che causi o subisca interazioni farmacocinetiche.

Di conseguenza, negli studi *in vivo* non sono state osservate interazioni farmacocinetiche clinicamente rilevanti tra pregabalin e fenitoina, carbamazepina, acido valproico, lamotrigina, gabapentin, lorazepam, ossicodone o etanolo. L’analisi farmacocinetica sulla popolazione ha indicato che antidiabetici orali, diuretici, insulina, fenobarbital, tiagabina e topiramato non hanno avuto un effetto clinicamente significativo sulla clearance del pregabalin.

La somministrazione concomitante di pregabalin con i contraccettivi orali noretisterone e/o etinilestradiolo non influenza la farmacocinetica delle due sostanze allo *steady-state*.

Dosi orali multiple di pregabalin somministrato con ossicodone, lorazepam o etanolo non hanno avuto effetti clinicamente importanti sulla respirazione. Sembra che pregabalin abbia un effetto additivo sulla compromissione della funzione cognitiva e sulla funzione motoria causate dall’ossicodone. Pregabalin può potenziare gli effetti di etanolo e lorazepam.

Non sono stati condotti specifici studi di interazione farmacodinamica in volontari sani anziani. Studi di interazione sono stati condotti solo negli adulti.

**4.6 Gravidanza ed allattamento**

Non sono disponibili dati adeguati sull’uso di pregabalin in donne in gravidanza.

Studi condotti sull’animale hanno dimostrato tossicità riproduttiva (vedere paragrafo 5.3). Non è noto il potenziale rischio per l’uomo. Lyrica non deve essere utilizzato durante la gravidanza a meno che non sia chiaramente necessario (se il beneficio per la madre è chiaramente superiore al potenziale rischio per il feto). Le donne in età fertile devono utilizzare un metodo di contraccezione efficace.

Non è noto se pregabalin sia escreto nel latte materno; tuttavia, è presente nel latte dei ratti. Pertanto, si raccomanda di non allattare durante il trattamento con pregabalin.

**4.7 Effetti sulla capacità di guidare veicoli e sull’uso di macchinari**

Lyrica può avere un’influenza minima o moderata sulla capacità di guidare e usare macchinari. Lyrica può causare capogiri e sonnolenza e pertanto può influenzare la capacità di guidare veicoli o usare macchinari. Si deve consigliare ai pazienti di non guidare, utilizzare macchinari complessi o intraprendere altre attività potenzialmente pericolose fino a quando non sarà noto se questo medicinale influenza la capacità di svolgere queste attività.

**4.8 Effetti indesiderati**

Il programma clinico di pregabalin ha coinvolto oltre .9000 pazienti trattati con pregabalin; di questi pazienti oltre 5.000 sono stati arruolati in studi clinici controllati in doppio cieco verso placebo. Le reazioni avverse segnalate più comunemente sono state capogiri e sonnolenza. Le reazioni avverse sono state di solito di intensità lieve-moderata. In tutti gli studi controllati, la percentuale di interruzione per reazioni avverse è stata del 13 % per i pazienti in trattamento con pregabalin e del 7 % per quelli in trattamento con placebo. Le reazioni avverse più comuni che hanno comportato l’interruzione del trattamento con pregabalin sono state capogiri e sonnolenza.

Nella tabella sottostante sono elencate tutte le reazioni avverse che si sono verificate con un’incidenza maggiore del placebo ed in più di un paziente e sono classificate per classe sistemica organica e per frequenza (molto comuni (>1/10), comuni (> 1/100, < 1/10), non comuni (> 1/1.000, < 1/100) e rari (<1/1.000). All’interno di ciascuna classe di frequenza, gli effetti indesiderati sono riportati in ordine di gravità decrescente.

Le reazioni avverse elencate possono essere associate anche alla malattia di base e/o all’impiego di farmaci concomitanti.

Nel trattamento del dolore neuropatico centrale dovuto ad una lesione della colonna vertebrale l’incidenza degli eventi avversi in generale, del SNC e della sonnolenza in particolare, è aumentata

(vedere paragrafo 4.4).

Altre reazioni segnalate durante la fase di commercializzazione del prodotto sono incluse con una frequenza non nota in corsivo nella lista sottostante

| **Disturbi del sistema immunitario** | |
| --- | --- |
| Frequenza non nota | *Ipersensibilità, reazione allergica* |
| **Patologie del sistema emolinfopoietico** | |
| Rari | *Neutropenia* |
| **Disturbi del metabolismo e della nutrizione** | |
| Comuni | *Aumento dell’appetito* |
| Non comuni | *Anoressia* |
| Rari | *Ipoglicemia* |
| **Disturbi psichiatrici** | |
| Comuni | *Euforia, confusione, irritabilità, riduzione della libido* |
| Non comuni | *Allucinazioni, attacchi di panico, irrequietezza, agitazione, depressione, umore depresso, alterazioni dell’umore, depersonalizzazione, insonnia, peggioramento dell’insonnia, difficoltà nel trovare le parole, sogni alterati, aumento della libido, anorgasmia, apatia* |
| Rari | *Disinibizione, innalzamento del tono dell’umore* |
| **Patologie del sistema nervoso** | |
| Molto comuni | *Capogiri, sonnolenza* |
| Comuni | *Atassia, coordinazione alterata, tremori, disartria, compromissione della memoria, disturbi dell’attenzione, parestesia* |
| Non comuni | *Sincope, stupor, mioclono, iperattività psicomotoria, disturbi del campo visivo, ageusia, discinesia, capogiri posturali, tremore intenzionale, nistagmo, disturbi cognitivi, alterazioni del linguaggio, iporiflessia, ipoestesia, amnesia, iperestesia, sensazione di bruciore* |
| Rari | *Ipocinesia, parosmia, disgrafia* |
| Frequenza non nota | *Cefalea* |
| **Patologie dell’occhio** | |
| Comuni | *Offuscamento della vista, diplopia* |
| Non comuni | *Disturbi della vista, gonfiore oculare, riduzione dell’acuità visiva, dolore oculare, astenopia, secchezza oculare, aumento della lacrimazione* |
| Rari | *Perdita della visione periferica, oscillopsia, alterata percezione della profondità visiva, fotopsia, irritazione oculare, midriasi, strabismo, luminosità visiva* |
| **Patologie dell'orecchio e del labirinto** | |
| Comuni | *Vertigini* |
| Rari | *Iperacusia* |
| **Patologie cardiache** | |
| Non comuni | *Tachicardia* |
| Rari | *Blocco atrioventricolare di primo grado, tachicardia sinusale, bradicardia sinusale, aritmia sinusale* |
| Frequenza non nota | *Scompenso cardiaco* |
| **Patologie vascolari** | |
| Non comuni | *Arrossamento, vampate di calore* |
| Rari | *Ipotensione, ipertensione, sensazione di freddo a livello periferico* |
| **Patologie respiratorie, toraciche e mediastiniche** | |
| Non comuni | *Dispnea, secchezza nasale* |
| Rari | *Epistassi, senso di costrizione alla gola, rinofaringite, tosse, congestione nasale, rinite, russare* |
| **Patologie gastrointestinali** | |
| Comuni | *Vomito, secchezza delle fauci, stipsi, flatulenza* |
| Non comuni | *Distensione addominale, malattia da reflusso gastroesofageo, ipersecrezione salivare, ipoestesia orale* |
| Rari | *Ascite, pancreatite, disfagia* |
| Frequenza non nota | *Gonfiore della lingua, diarrea, nausea* |
| **Patologie della cute e del tessuto sottocutaneo** | |
| Non comuni | *Rash papulare, sudorazione* |
| Rari | *Orticaria, sudori freddi* |
| Frequenza non nota | *Prurito* |
| **Patologie dell’apparato muscoloscheletrico e tessuto connettivo** | |
| Non comuni | *Contrazioni muscolari, gonfiore della articolazioni, crampi muscolari, mialgia, artralgia, dolore alla schiena, dolore agli arti, rigidità muscolare* |
| Rari | *Rabdomiolisi, spasmi del tratto cervicale, dolore al collo* |
| **Patologie renali e urinarie** | |
| Non comuni | *Incontinenza urinaria, disuria* |
| Rari | *Insufficienza renale, oliguria* |
| **Patologie dell'apparato riproduttivo e della mammella** | |
| Comuni | *Disfunzione erettile* |
| Non comuni | *Ritardo nell’eiaculazione, disfunzione sessuale* |
| Rari | *Amenorrea, secrezione mammaria, dolore al seno, dismenorrea, ipertrofia del seno* |
| **Patologie sistemiche e condizioni relative alla sede di somministrazione** | |
| Comuni | *Anomalie dell’andatura, sensazione di stato di ebbrezza, spossatezza, edema periferico, edema,* |
| Non comuni | *Cadute, costrizione del torace, astenia, sete* |
| Rari | *Anasarca, piressia, brividi, peggioramento del dolore* |
| Frequenza non nota | *Edema facciale* |
| **Esami diagnostici** | |
| Comuni | *Aumento di peso* |
| Non comuni | *Aumento della alanina-aminotransferasi, aumento della creatinfosfochinasi, aumento dell’aspartato aminotransferasi, riduzione della conta piastrinica* |
| Rari | *Aumento della glicemia, riduzione della potassiemia, riduzione del numero dei globuli bianchi, aumento della creatinina ematica, riduzione di peso* |

In alcuni pazienti, a seguito della sospensione di trattamenti a breve e a lungo termine con pregabalin sono stati osservati sintomi da sospensione. Sono stati segnalati i seguenti eventi: insonnia, cefalea, nausea, diarrea, sindrome influenzale, nervosismo, depressione, dolore, sudorazione e capogiri. I pazienti devono essere informati di questa evenienza prima dell’inizio del trattamento.

Per quanto concerne la sospensione del trattamento a lungo termine con pregabalin, non vi sono dati sull’incidenza e sulla gravità dei sintomi da sospensione in relazione alla durata del trattamento ed al dosaggio di pregabalin.

**4.9 Sovradosaggio**

Con sovradosaggi fino a 15 g, non sono state segnalate reazioni avverse impreviste. Il trattamento del sovradosaggio di pregabalin deve includere misure generali di supporto e, se necessario, può includere l’emodialisi (vedere paragrafo 4.2 Tabella 1).

**5. PROPRIETÀ FARMACOLOGICHE**

**5.1 Proprietà farmacodinamiche**

Categoria farmacoterapeutica: Antiepilettici, codice ATC: N03AX16

Il principio attivo, pregabalin, è un analogo ((S-3-(aminometil)-5-acido metilesanoico) dell’acido gamma-aminobutirrico.

Meccanismo d’azione

Pregabalin si lega alla subunità accessoria (proteina α_2_-δ) dei canali del calcio voltaggio dipendenti nel sistema nervoso centrale, con possibilità di spiazzare il gabapentin-[^3^H].

Esperienza clinica

*Dolore neuropatico*

L’efficacia è stata dimostrata in studi sulla neuropatia diabetica, sulla nevralgia post-erpetica e sulle lesioni della colonna vertebrale. L’efficacia non è stata studiata in altri modelli di dolore neuropatico.

Pregabalin è stato studiato in 10 studi clinici controllati, nei quali è stato somministrato 2 volte al giorno (BID) per un periodo fino a 13 settimane e 3 volte al giorno (TID) per un periodo fino a 8 settimane. Nel complesso, i profili di sicurezza e di efficacia per i regimi posologici BID e TID sono stati simili.

Negli studi clinici fino a 12 settimane su entrambi il dolore neuropatico periferico e centrale è stata osservata una riduzione del dolore dopo una settimana di trattamento e tale riduzione si è mantenuta per tutta la durata del trattamento.

Negli studi clinici controllati sul dolore neuropatico periferico il 35 % dei pazienti trattati con pregabalin ed il 18 % di quelli in trattamento con placebo ha riportato un miglioramento del 50 % nella scala del dolore. Nei pazienti che non hanno riportato sonnolenza, questo miglioramento è stato osservato nel 33 % dei pazienti trattati con pregabalin e nel 18 % di quelli in trattamento con placebo. La percentuale di risposta per i pazienti che hanno riportato sonnolenza è stata del 48 % per i pazienti trattati con pregabalin e del 16 % per quelli trattati con placebo.

Nello studio clinico controllato sul dolore neuropatico centrale, il 22% dei pazienti trattati con Pregabalin ed il 7% di quelli che assumevano placebo hanno riportato un miglioramento del 50% nella scala del dolore.

*Epilessia*

Pregabalin è stato studiato in 3 studi clinici controllati della durata di 12 settimane sia con due (BID) che con tre (TID) somministrazione giornaliere. Nel complesso, i profili di sicurezza e di efficacia per i regimi posologici BID o TID sono stati simili.

E’ stata osservata una riduzione nella frequenza degli attacchi epilettici entro una settimana di trattamento.

*Disturbo d’Ansia Generalizzata*

Pregabalin è stato studiato in 6 studi clinici controllati della durata di 4-6 settimane, in uno studio su soggetti anziani della durata di 8 settimane ed in uno studio a lungo termine, con una fase di 6 mesi in doppio cieco, sulla prevenzione delle ricadute.

Entro una settimana di trattamento è stata osservata un’attenuazione dei sintomi del Disturbo d’Ansia Generalizzata della Scala di Hamilton per l’Ansia (HAM-A).

Negli studi clinici controllati (durata 4-8 settimane), il 52% dei pazienti trattati con pregabalin ed il 38% di quelli del gruppo placebo hanno riportato un miglioramento di almeno il 50% nel punteggio totale della scala HAM-A dal basale alla fine dello studio.

**5.2 Proprietà farmacocinetiche**

La farmacocinetica di pregabalin allo steady-state è simile nei volontari sani, nei pazienti epilettici in trattamento con farmaci antiepilettici e nei pazienti con dolore cronico.

Assorbimento:

Pregabalin viene rapidamente assorbito quando somministrato a digiuno, con concentrazioni plasmatiche di picco raggiunte entro 1 ora dalla somministrazione di una dose singole o di dosi multiple. La biodisponibilità orale di pregabalin è ≥ 90% ed è indipendente dalla dose. A seguito di somministrazioni ripetute, lo steady-state viene raggiunto entro 24-48 ore. Il tasso di assorbimento di pregabalin diminuisce quando viene somministrato insieme al cibo, con una riduzione della C_max_ di circa il 25-30 % ed un ritardo nel t_max_ di circa 2.5 ore. Tuttavia, la somministrazione di pregabalin con il cibo non ha un effetto clinicamente significativo sull’assorbimento di pregabalin.

Distribuzione:

Negli studi preclinici, è stato dimostrato che pregabalin attraversa la barriera ematoencefalica nei topi, ratti e scimmie. E’ stato dimostrato che pregabalin attraversa la placenta nei ratti ed è presente nel latte dei ratti che allattano. Nell’uomo, il volume apparente di distribuzione di pregabalin a seguito di somministrazione orale è di circa 0.56 l/kg. Pregabalin non si lega alle proteine plasmatiche.

Metabolismo:

Pregabalin viene metabolizzato nell’uomo in modo trascurabile. In seguito alla somministrazione di una dose di pregabalin radiomarcato, circa il 98 % della radioattività riscontrata nelle urine era presente sotto forma di farmaco immodificato. Il derivato N-metilato del pregabalin, il principale metabolita del pregabalin riscontrato nelle urine, corrisponde allo 0.9 % della dose. Negli studi preclinici, non vi è stata un’indicazione di racemizzazione del pregabalin S-enantiomero in R-enantiomero.

Eliminazione:

Pregabalin viene eliminato dalla circolazione principalmente attraverso escrezione renale sotto forma di farmaco immodificato. L’emivita media di eliminazione di pregabalin è di 6.3 ore. La clearance plsmatica e la clearance renale sono direttamente proporzionali alla clerance della creatinina (vedere paragrafo 4.2 Compromissione renale).

In pazienti con ridotta funzionalità renale o sottoposti ad emodialisi è necessario un aggiustamento posologico (vedere paragrafo 4.2 Tabella 1).

Linearità / non linearità:

La farmacocinetica di pregabalin è lineare nell’ambito del range posologico giornaliero raccomandato. La variabilità nella farmacocinetica fra i soggetti è bassa (< 20%). La farmacocinetica a seguito di dosi multiple è prevedibile dai dati in dose singola. Pertanto, non è necessario un monitoraggio di routine delle concentrazioni plasmatiche di pregabalin.

Farmacocinetica in gruppi particolari di pazienti

Sesso

Gli studi clinici indicano che il sesso non influisce in modo clinicamente significativo sulle concentrazioni plasmatiche di pregabalin.

Compromissione renale

La clearance di pregabalin è direttamente proporzionale alla clearance della creatinina. Inoltre, pregabalin viene rimosso in maniera efficace dal plasma mediante emodialisi (dopo una seduta di emodialisi di 4 ore le concentrazioni plasmatiche di pregabalin si riducono di circa il 50 %). Poiché l’eliminazione renale rappresenta la principale via di eliminazione, nei pazienti con compromissione renale è necessaria una riduzione della dose e dopo una seduta di emodialisi è necessaria la somministrazione di una dose supplementare (vedere paragrafo 4.2 Tabella 1).

Compromissione epatica

Non sono stati condotti studi specifici di farmacocinetica in pazienti con compromissione della funzionalità epatica. Poiché pregabalin non viene metabolizzato in modo significativo e viene escreto principalmente sotto forma di farmaco immodificato nelle urine, la compromissione della funzionalità epatica non dovrebbe alterare significativamente le concentrazioni plasmatiche di pregabalin.

Anziani (età superiore ai 65 anni)

La clearance di pregabalin tende a diminuire con l’aumentare dell’età. Questa riduzione nella clearance di pregabalin somministrato per via orale è in linea con le riduzioni della clearance della creatinina associate all’aumentare dell’età. In pazienti che presentano una compromissione della funzionalità renale correlata all’età può essere necessaria una riduzione della dose di pregabalin (vedere paragrafo 4.2 Tabella 1).

**5.3 Dati preclinici di sicurezza**

Sulla base di studi convenzionali di farmacologia di sicurezza nell’animale, pregabalin è stato ben tollerato alle dosi clinicamente significative. Negli studi di tossicità per somministrazioni ripetute nel ratto e nella scimmia, sono stati osservati effetti sul Sistema Nervoso Centrale inclusi ipoattività, iperattività e atassia. Nel ratto albino anziano è stato comunemente osservato un aumento di incidenza dell’atrofia retinica a seguito dell’esposizione a lungo termine al pregabalin con un’esposizione ≥ 5 volte l’esposizione media nell’uomo alle massime dosi cliniche raccomandate.

Pregabalin non si è dimostrato teratogeno nel topo, nel ratto o nel coniglio. Nei ratti e nei conigli si è verificata tossicità fetale solo con esposizioni sufficientemente al di sopra dell’esposizione nell’uomo. Negli studi di tossicità prenatale/postnatale, pregabalin ha indotto tossicità nello sviluppo della prole nei ratti ad un’esposizione > 2 volte l’esposizione umana massima raccomanda.

Pregabalin non si è dimostrato genotossico sulla base dei risultati di una serie di test *in vitro* ed *in vivo*.

Sono stati condotti studi di carcinogenesi a due anni con pregabalin in ratti e topi. Non è stata osservata formazione di tumori nei ratti esposti a dosi fino a 24 volte superiori l’esposizione media nell’uomo alla massima dose clinica raccomandata di 600mg/die. Nei topi, non è stato osservato un aumento di incidenza dei tumori con esposizioni simili all’esposizione media nell’uomo, ma è stato osservato un aumento di incidenza di emangiosarcoma con esposizioni più elevate. Il meccanismo non genotossico della formazione di tumori indotta da pregabalin nei topi causa alterazioni piastriniche ed un’associata proliferazione delle cellule endoteliali. Queste alterazioni piastriniche non sono state riscontrate nei ratti o nell’uomo sulla base dei dati clinici limitati a breve ed a lungo termine. Non vi è evidenza per suggerire un rischio associato nell’uomo.

Nei ratti giovani i tipi di tossicità non differiscono qualitativamente da quelli osservati nei ratti adulti. Tuttavia, i ratti giovani sono più sensibili. Alle esposizioni terapeutiche, si sono evidenziati segni clinici a carico del Sistema Nervoso Centrale di iperattività e bruxismo ed alcuni cambiamenti nella crescita (riduzione transitoria dell’aumento del peso corporeo). Sono stati osservati effetti sul ciclo mestruale con 5 volte l’esposizione terapeutica nell’uomo. Una riduzione della risposta agli stimoli acustici è stata osservata nei ratti giovani 1-2 settimane dopo un’esposizione 2 volte maggiore l’esposizione terapeutica umana. Nove settimane dopo l’esposizione, questo effetto non è stato più osservato.

**6. INFORMAZIONI FARMACEUTICHE**

**6.1 Elenco degli eccipienti**

Contenuto della capsula: Lattosio monoidrato Amido di mais Talco Opercolo della capsula: Gelatina Diossido di itanio (E171) Sodio laurilsolfato Silice colloidale anidra Acqua purificata Ossido di ferro rosso (E172) (capsule da 75 mg, 100 mg, 200 mg, 225 mg, 300 mg). Inchiostro: Shellac Ossido di ferro nero (E172) Glicole ropilenico

Potassio idrossido

**6.2 Incompatibilità**

Non pertinente.

**6.3 Periodo di validità**

3 anni.

**6.4 Precauzioni particolari per la conservazione**

Questo medicinale non richiede alcuna speciale condizione di conservazione.

**6.5 Natura e contenuto del contenitore**

LYRICA 25 mg capsule rigide:

Blister in PVC/Alluminio contenenti 14, 21, 56, 84 o 112 (2 x 56) capsule rigide.

100 x 1 capsule rigide in blister divisibili per dose unitaria in PVC/Alluminio.

LYRICA 50 mg capsule rigide:

Blister in PVC/Alluminio contenenti 14, 21, 56 o 84 capsule rigide.

100 x 1 capsule rigide in blister divisibili per dose unitaria in PVC/Alluminio.

LYRICA 75 mg capsule rigide:

Blister in PVC/Alluminio contenenti 14, 56 o 112 (2 x 56) capsule rigide.

100 x 1 capsule rigide in blister divisibili per dose unitaria in PVC/Alluminio.

LYRICA 100 mg capsule rigide:

Blister in PVC/Alluminio contenenti 21 o 84 capsule rigide.

100 x 1 capsule rigide in blister divisibili per dose unitaria in PVC/Alluminio.

LYRICA 150 mg capsule rigide:

Blister in PVC/Alluminio contenenti 14, 56 o 112 (2 x 56) capsule rigide.

100 x 1 capsule rigide in blister divisibili per dose unitaria in PVC/Alluminio.

Flacone HDPE contenente 200 capsule rigide.

LYRICA 200 mg capsule rigide:

Blister in PVC/Alluminio contenenti 21 o 84 capsule rigide.

100 x 1 capsule rigide in blister divisibili per dose unitaria in PVC/Alluminio.

LYRICA 225 mg capsule rigide:

Blister in PVC/Alluminio contenenti 14 o 56 capsule rigide.

100 x 1 capsule rigide in blister divisibili per dose unitaria in PVC/Alluminio.

LYRICA 300 mg capsule rigide:

Blister in PVC/Alluminio contenenti 14, 56 o 112 (2 x 56) capsule rigide.

100 x 1 capsule rigide in blister divisibili per dose unitaria in PVC/Alluminio.

Flacone HDPE contenente 200 capsule rigide.

E’ possibile che non tutte le confezioni siano commercializzate.

**6.6 Precauzioni particolari per lo smaltimento e la manipolazione**

Nessuna istruzione particolare.

**7. TITOLARE DELL'AUTORIZZAZIONE ALL'IMMISSIONE IN COMMERCIO**

Pfizer Limited,

Ramsgate Road,

Sandwich,

Kent

CT13 9NJ

Regno Unito

**8. NUMERO(I) DELL’AUTORIZZAZIONE ALL’IMMISSIONE IN COMMERCIO**

LYRICA 25 mg capsule rigide:

EU/1/04/279/001-005

EU/1/04/279/026

LYRICA 50 mg capsule rigide

EU/1/04/279/006-010

LYRICA 75 mg capsule rigide

EU/1/04/279/011-013

EU/1/04/279/027

EU/1/04/279/030

LYRICA 100 mg capsule rigide

EU/1/04/279/014-016

LYRICA 150 mg capsule rigide

EU/1/04/279/017-019

EU/1/04/279/028

EU/1/04/279/031

LYRICA 200 mg capsule rigide

EU/1/04/279/020-022

LYRICA 200 mg capsule rigide

EU/1/04/279/020-022

LYRICA 225 mg capsule rigide

EU/1/04/279/033-035

LYRICA 300 mg capsule rigide

EU/1/04/279/023-025

EU/1/04/279/029

EU/1/04/279/032

**9. DATA DELLA PRIMA AUTORIZZAZIONE/ RINNOVO DELL’AUTORIZZAZIONE**

Data della prima autorizzazione: 06/07/2004

Data dell’ultimo rinnovo:

**10. DATA DI REVISIONE DEL TESTO**

17/01/2007

Allegato Protocollo 3

Descrizione delle caratteristiche di Oxycontin®

**1. DENOMINAZIONE DELLA SPECIALITÀ MEDICINALE**

OxyContin 10 mg compresse a rilascio prolungato

OxyContin 20 mg compresse a rilascio prolungato

OxyContin 40 mg compresse a rilascio prolungato

OxyContin 80 mg compresse a rilascio prolungato

**2. COMPOSIZIONE QUALITATIVA E QUANTITATIVA**

Una compressa di OxyContin 10 mg contiene 9 mg di ossicodone

pari a 10 mg di ossicodone cloridrato.

Una compressa di OxyContin 20 mg contiene 18 mg di ossicodone

pari a 20 mg di ossicodone cloridrato.

Una compressa di OxyContin 40 mg contiene 36 mg di ossicodone

pari a 40 mg di ossicodone cloridrato.

Una compressa di OxyContin 80 mg contiene 72 mg di ossicodone

pari a 80 mg di ossicodone cloridrato

Per gli eccipienti vedere sezione 6.1

**3. FORMA FARMACEUTICA**

Compresse a rilascio prolungato, rotonde, convesse.

Le compresse da 10 mg sono bianche,

con OC impresso su una faccia e 10 sull’altra.

Le compresse da 20 mg sono rosa,

con OC impresso su una faccia e 20 sull’altra.

Le compresse da 40 mg sono gialle,

con OC impresso su una faccia e 40 sull’altra.

Le compresse da 80 mg sono verdi,

con OC impresso su una faccia e 80 sull’altra.

**4. INFORMAZIONI CLINICHE**

**4.1 Indicazioni terapeutiche.**

Trattamento del dolore intenso.

**4.2 Posologia e modo di somministrazione.**

Le compresse di OxyContin vanno deglutite intere, e non devono essere rotte, masticate o frantumate. Assumendo le compresse di OxyContin rotte, masticate o frantumate si ottiene un rapido rilascio e assorbimento della dose potenzialmente letale di ossicodone. Adulti e anziani: le compresse di OxyContin vanno assunte a intervalli di 12 ore. Il dosaggio dipende dall’intensità del dolore, dal bisogno di analgesici manifestato in passato dal paziente, dal peso corporeo e dal sesso del paziente (nelle donne si producono concentrazioni plasmatiche più elevate). La dose iniziale usuale per i pazienti anziani debilitati, per i pazienti non dipendenti da oppiacei o per i pazienti che presentano dolore intenso non controllabile con oppiacei più deboli, è di 10 mg ogni 12 ore. Per ottenere sollievo dal dolore il dosaggio deve poi essere titolato accuratamente, tutti i giorni se necessario. Quando è possibile i dosaggi vanno aumentati in incrementi del 25%-50%. Il dosaggio che controlla il dolore ed è ben tollerato per un periodo di 12 ore rappresenta il dosaggio esatto per quel paziente. Quando per ottenere il sollievo dal dolore si manifesta la necessità di una somministrazione del medicinale con una frequenza superiore a due volte al giorno, ciò indica che il dosaggio delle compresse di OxyContin deve essere aumentato. Conversione dalla morfina orale: nei pazienti trattati con morfina orale prima del trattamento con OxyContin la dose giornaliera deve basarsi sul rapporto che segue: 10 mg di ossicodone orale equivalgono a 20 mg di morfina orale. Occorre sottolineare che questo rapporto serve da guida nel determinare la dose necessaria di compresse di OxyContin. La variabilità fra pazienti esige che la dose esatta sia calcolata e adattata accuratamente per ogni paziente. Pazienti anziani: studi farmacocinetici controllati in pazienti anziani (sopra i 65 anni) hanno rivelato che, rispetto agli adulti più giovani, la clearance dell’ossicodone è solo leggermente ridotta. In base all’età non sono state osservate reazioni avverse infauste dovute al farmaco; pertanto, negli anziani, sono adeguati le dosi e gli intervalli di dosaggio impiegati per gli adulti.

Dolore non maligno: il trattamento con OxyContin deve essere di breve durata e intermittente per minimizzare il rischio di dipendenza. La necessità di continuare il trattamento deve essere valutata ad intervalli regolari. I pazienti, di solito, non dovrebbero avere bisogno di più di 160 mg al giorno.

Dolore oncologico: nei pazienti la dose deve essere aggiustata fino a raggiungere quella che produce sollievo dal dolore salvo che ciò non sia impedito dalla comparsa di incontrollabili reazioni avverse dovute al farmaco.

Pazienti con insufficienza renale o epatica: a differenza delle preparazioni di morfina, la somministrazione di compresse di OxyContin non produce livelli significativi di metaboliti attivi. Comunque, in questa popolazione di pazienti, la concentrazione plasmatica di ossicodone potrebbe aumentare rispetto ai pazienti con una normale funzione renale e epatica. Pertanto, occorre scegliere la dose iniziale in questi pazienti con cautela, e cioè, da un terzo a metà della dose usuale, scegliendo con cura la dose opportuna. Nel caso di insufficienza epatica grave, potrebbe anche essere necessario ridurre la frequenza del dosaggio. Non ci sono dati sull’uso di OxyContin in pazienti sottoposti a emodialisi. Adulti sotto i 20 anni e bambini: non consigliato.

**4.3 Controindicazioni.**

Ipersensibilità nota verso l’ossicodone, morfina e altri farmaci oppiacei, e verso gli altri componenti del prodotto. Depressione respiratoria, trauma cranico, ileo paralitico, addome acuto, svuotamento gastrico ritardato, affezione ostruttiva grave delle vie respiratorie, asma bronchiale grave, ipercapnia, affezione epatica acuta, somministrazione concomitante di inibitori della monoaminoossidasi o entro 2 settimane dalla sospensione del loro uso. La sicurezza sull’uso preoperatorio e fino a 24 ore postoperatorio di OxyContin non è stata stabilita e non può essere consigliata.

**4.4 Speciali avvertenze e precauzioni per l’uso.**

Come per tutti i narcotici, è consigliabile ridurre il dosaggio in caso di ipotiroidismo. Usare con cautela nei pazienti con una dipendenza dagli oppioidi, pazienti con psicosi tossica e nei pazienti con elevata pressione intracranica, ipotensione, ipovolemia, affezione delle vie biliari, pancreatite, alterazione infiammatoria intestinale, ipertrofia prostatica, insufficienza corticosurrenale, alcoolismo acuto, affezione cronica renale ed epatica, e nei pazienti debilitati. Le compresse di OxyContin non devono essere somministrate nei casi in cui vi è la possibilità che si verifichi un ileo paralitico. L’attività di OxyContin 80 mg compresse può causare una depressione respiratoria letale quando è assunto da pazienti non precedentemente esposti ad oppiodi. Se durante l’uso si dovesse sospettare o si verificasse un ileo paralitico, la somministrazione di compresse di OxyContin deve essere immediatamente sospesa. Così come per tutte le preparazioni di oppiacei, non bisogna somministrare compresse di OxyContin per 24 ore prima dell’intervento a quei pazienti che devono essere sottoposti a cordotomia o altri interventi chirurgici per alleviare il dolore. Se, successivamente, le condizioni del paziente suggeriscono un trattamento con compresse di OxyContin, allora occorre regolare il dosaggio in base alle sopravvenute esigenze postoperatorie. Come per tutte le preparazioni oppiacee, dopo un intervento chirurgico addominale occorre usare cautela nel somministrare compresse di OxyContin poiché è noto che gli oppiacei alterano la motilità intestinale e non devono essere usati fino a quando il medico non è certo della presenza di una funzione intestinale normale. I pazienti trattati con ossicodone possono andare incontro a dipendenza fisica. La sospensione improvvisa del farmaco o la somministrazione di un antagonista oppiaceo potrebbe provocare una sindrome da astinenza. Quando non è più necessaria la terapia con compresse di OxyContin, pazienti trattati con dosi di 20-60 mg/die possono generalmente interrompere la terapia improvvisamente senza problemi. I dosaggi più elevati devono essere sospesi nell’arco di alcuni giorni, riducendo la dose giornaliera di circa il 50% per i primi due giorni, e in seguito del 25% ogni due giorni fino a quando la dose totale giornaliera raggiunga la dose raccomandata per i pazienti non dipendenti dagli oppiacei, e cioè 10 mg ogni 12 ore. Si può quindi interrompere il trattamento. Le compresse di OxyContin da 80 mg vanno somministrate soltanto a pazienti che tollerano gli oppiacei e che richiedono dosaggi giornalieri di ossicodone di 160 mg o più. Occorre essere cauti nel prescrivere compresse di tale concentrazione. Le compresse contengono le seguenti quantità di lattosio: 69,25 mg (per la compressa da 10 mg); 59,25 mg (per la compressa da 20 mg); 35,25 mg (per la compressa da 40 mg); 78,50 mg (per la compressa da 80 mg).

**4.5 Interazioni con altri medicamenti e altre forme di interazioni.**

Occorre usare cautela nella somministrazione di OxyContin, e occorre ridurne il dosaggio, nei pazienti che assumono depressivi del sistema nervoso centrale poiché gli oppiacei potenziano gli effetti di fenotiazine, antidepressivi triciclici, anestetici, ipnotici, sedativi, alcool, miorilassanti e antiipertensivi. È noto che gli inibitori della monoamino-ossidasi interagiscono con gli analgesici narcotici dando origine a eccitazione o depressione del SNC con crisi ipertensive o ipotensive. L’ossicodone è metabolizzato dal sistema enzimatico citocromo P450, ma una valutazione completa delle interazioni con altri farmaci metabolizzati attraverso questa via non è stata intrapresa. La somministrazione concomitante di chinidina, un inibitore del citocromo P450-2D6, ha determinato un aumento del Cmax dell’ossicodone dell’11%, dell’AUC del 13% e del t1/2 elim del 14%; è stato osservato anche un aumento del livello del norossicodone. Gli effetti farmacodinamici di OxyContin non sono stati alterati. Questa interazione può essere osservata per altri potenti inibitori dell’enzima citocromo P450-2D6. Il metabolismo dell’ossicodone può essere inibito dalla cimetidina e dagli inibitori o substrati del citocromo P450-3A4 come ketoconazolo ed eritromicina.

**4.6 Uso in gravidanza e allattamento.**

Si sconsiglia l’uso di compresse di OxyContin in gravidanza. L’ossicodone è secreto nel latte materno e può provocare depressione respiratoria nel neonato. Pertanto, le compresse di OxyContin non devono essere assunte dalle mamme che allattano.

**4.7 Effetti sulla capacità di guidare e sull’uso di macchine.**

L’ossicodone può modificare le reazioni dei pazienti in misura diversa in funzione del dosaggio e della suscettibilità individuale. Se colpiti i pazienti non devono guidare automobili o azionare macchinari.

**4.8 Effetti indesiderati.**

Le reazioni avverse più comuni osservate sono nausea e stipsi e si verificano in circa il 25-30% dei pazienti. Se la nausea e il vomito sono fastidiosi si può associare all’ossicodone un antiemetico. Come per tutti i forti oppiacei, la stipsi deve essere prevista e deve essere trattata con lassativi adeguati. Se gli eventi avversi connessi agli oppiacei dovessero persistere devono essere ricercate altre cause. Le reazioni avverse dovute al farmaco sono tipiche degli agonisti completi degli oppiacei e tendono a diminuire con il passare del tempo, eccetto che per la stipsi. La previsione delle reazioni avverse dovute al farmaco e la gestione adeguata del paziente possono migliorarne l’accettabilità. La reazione avversa più grave, così come per gli altri oppiacei, è la depressione respiratoria (vedere paragrafo "Sovradosaggio"). È più probabile che questa reazione si manifesti negli anziani, e nei pazienti debilitati o intolleranti agli oppiacei. Le reazioni avverse osservate durante le sperimentazioni cliniche sono state:

Molto comuni (>10%)

Apparato digestivo: Stipsi, vomito, nausea.

Sistema nervoso: Sonnolenza, capogiri.

Derma e appendici Prurito.

Comuni (1-10%)

Generali: Edema, febbre, dolori addominali, astenia, cefalee.

Sistema cardiovascolare: Vasodilatazione.

Apparato digestivo: Anoressia, diarrea, secchezza della fauci, dispepsia, flatulenza.

Sistema nervoso: Sogni anomali, ansia, confusione, depressione, insonnia, allucinazioni, **debolezza.**

Apparato respiratorio: Dispnea, **broncospasmo.**

Derma e appendici: Irritazioni, sudorazione.

Apparato urogenitale: Alterazione della minzione.

Insoliti (0,1 - 1%)

Generali: Brividi, dolore toracico, **reazioni allergiche.**

Sistema cardiovascolare: Palpitazioni, sincope.

Apparato digestivo: Disfagia, gastrite, ulcerazione della bocca, eruttazione, ileo, stomatite, **spasmi biliari.**

Sistema endocrino: Sindrome da inappropriata secrezione dell'ormone antidiuretico.

Metabolismo e nutrizione: Disidratazione, alterazione del peso.

Sistema nervoso: Andatura anormale, amnesia, depersonalizzazione, euforia, ipercinesia, ipertonia, ipoestesia, ipotonia, alterazione della parola, stupore, tremore, spasmo muscolare, vertigini, convulsioni epilettiche, variazione dell'umore, parestesie, alterazione del pensiero, sindrome da sospensione, diminuzione della libido.

Apparato respiratorio: Rinite, epistassi, singhiozzo, alterazione

della voce.

Derma ed appendici: Pelle secca.

Sensi: Alterazione della lacrimazione, alterazione della visione, alterazione del gusto, tinnito, **miosi.**

Apparato urogenitale: Ritenzione urinaria, amenorrea, impotenza.

Nei pazienti trattati con OxyContin può verificarsi una tolleranza sebbene ciò non abbia costituito un problema rilevante durante il programma di sperimentazione clinica. Quei pazienti che hanno avuto bisogno di un aumento di dosaggio marcato devono essere sottoposti a una valutazione accurata del loro schema terapeutico per il controllo del dolore. La sospensione improvvisa delle compresse di OxyContin o la somministrazione di un antagonista oppiaceo può dare origine a una sindrome da astinenza caratterizzata da ansietà, irritabilità, brividi, vampate di calore, piloerezione, dolore alle articolazioni, rinorrea, sudorazione, crampi addominali e diarrea. Se lo schema terapeutico per la riduzione del dosaggio suggerito al paragrafo 4.4 produce una sindrome da astinenza, occorre aumentare leggermente la dose fino a che non spariscono i segnali e i sintomi. Quindi occorre iniziare di nuovo a ridurre la dose e interporre periodi di tempo più lunghi fra una riduzione e l’altra.

**4.9 Sovradosaggio.**

I segnali di tossicità e sovradosaggio di ossicodone sono pupille fortemente miotiche, depressione respiratoria e ipotensione. Nei casi più gravi si possono verificare insufficienza circolatoria e coma profondo. Trattamento del sovradosaggio di ossicodone: bisogna rivolgere soprattutto attenzione a ripristinare un adeguato scambio respiratorio

liberando le vie respiratorie e istituendo una ventilazione assistita o controllata. Nel sovradosaggio grave somministrare 0,8 mg di nalossone per via endovenosa. Ripetere a intervalli di 2-3 minuti secondo le necessità, oppure mediante infusione di 2 mg in 500 ml di normale soluzione salina o al 5% di destrosio (0,004 mg/ml). La velocità dell’infusione deve essere regolata in rapporto alle precedenti dosi somministrate in bolo, e in funzione della risposta del paziente. In ogni modo, poiché la durata dell’azione del nalossone è relativamente breve, occorre monitorare attentamente il paziente fino a quando la respirazione spontanea non è chiaramente ristabilita. Le compresse di OxyContin continuano a rilasciare ossicodone e ad aumentare la dose assorbita fino a 12 ore dopo la somministrazione e la gestione del sovradosaggio di ossicodone deve essere adattata a questo comportamento del medicinale nell’organismo. In caso di sovradosaggio meno grave, somministrare 0,2 mg di nalossone per via endovenosa e, se necessario, continuare con incrementi di 0,1 mg ogni 2 minuti.

Non bisogna somministrare il nalossone in assenza di depressione respiratoria o circolatoria clinicamente significativa risultante dal sovradosaggio di ossicodone. Il nalossone deve essere somministrato con cautela alle persone che sono, o si sospetta siano, fisicamente dipendenti dall’ossicodone. In questi casi un’inversione improvvisa o completa degli effetti oppiacei potrebbe far precipitare il dolore e una sindrome da astinenza acuta. Potrebbe essere necessario svuotare il contenuto gastrico perché ciò può essere utile nel rimuovere il farmaco non assorbito, in modo particolare se è stata assunta una formulazione a rilascio prolungato.

**5. PROPRIETÀ FARMACOLOGICHE**

**5.1 Proprietà farmacodinamiche.**

L’ossicodone è un agonista oppiaceo senza proprietà antagoniste e ha un’affinità per i recettori oppiacei ,  e  del cervello e del midollo spinale. L’effetto terapeutico è principalmente analgesico, ansiolitico e sedativo.

Non è stato ancora pienamente caratterizzato l’esatto meccanismo d’azione.

**5.2 Proprietà farmacocinetiche.**

L’ossicodone ha un’elevata biodisponibilità assoluta che arriva all’87% dopo una somministrazione orale. Ha una emivita di eliminazione di circa 3 ore ed è metabolizzato principalmente in norossicodone e ossimorfone. L’ossimorfone ha una certa attività analgesica ma la sua concentrazione plasmatica è bassa e pertanto non contribuisce all’effetto farmacologico dell’ossicodone. Il rilascio dell’ossicodone dalle compresse di OxyContin avviene in due fasi con un rilascio iniziale relativamente veloce che produce un’insorgenza di effetto analgesico precoce seguito da un rilascio più controllato che determina la durata d’azione di 12 ore. L’emivita di eliminazione media apparente di OxyContin è di 4,5 ore, il che porta al raggiungimento dello stato stazionario nel tempo di circa un giorno. Il rilascio dell’ossicodone dalle compresse di OxyContin è indipendente dal pH. Le compresse di OxyContin hanno una biodisponibilità orale comparabile a quella dell’ossicodone orale tradizionale, ma il primo raggiunge le massime concentrazioni plasmatiche in circa 3 ore invece che da 1 a 1,5 ore circa. Le massime e le minime concentrazioni di ossicodone ottenute dalle compresse di OxyContin da 10 mg somministrate a intervalli di 12 ore sono equivalenti a quelle ottenute da 5 mg di ossicodone tradizionale somministrato ogni 6 ore. Le compresse di OxyContin da 10 mg, 20 mg, 40 mg e 80 mg sono bioequivalenti sia in termini di velocità che di grado di assorbimento. L’ingestione di un pasto standard ad alto contenuto di grassi non modifica la concentrazione massima di ossicodone o il grado di assorbimento di ossicodone dalle compresse di OxyContin.

**5.3 Dati preclinici sulla sicurezza.**

L’ossicodone non determina anormalità nei ratti in dosi fino a 8 mg/kg/die, o in conigli in dosi fino a 5 mg/kg/die. Non sono stati condotti studi sulla fertilità, pre e post-natali. I test in vitro mostrano che l’ossicodone è potenzialmente clastogenico ma non vi sono stati tali risultati nei test in vivo, nemmeno a dosi tossiche. Non sono stati condotti test sugli animali per la valutazione del potenziale carcinogenico dell’ossicodone a causa della lunga esperienza clinica con il farmaco.

**6. INFORMAZIONI FARMACEUTICHE**

**6.1 Lista degli eccipienti.**

OxyContin 10 mg compresse a rilascio prolungato - Nucleo: Lattosio, Polivinilpirrolidone, Ammoniometacrilato copolimero in dispersione, Triacetina, Alcool stearilico, Talco, Magnesio stearato, Rivestimento: Ipromellosa (E464), Idrossipropilcellulosa, Titanio biossido (E171), Macrogol. OxyContin 20 mg compresse a rilascio prolungato: Nucleo: Lattosio, Polivinilpirrolidone, Ammoniometacrilato copolimero in dispersione, Triacetina, Alcool stearilico, Talco, Magnesio stearato, Rivestimento: Ipromellosa (E464), Titanio biossido (E171), Macrogol, Polisorbato 80, Ossido di ferro (E172). OxyContin 40 mg compresse a rilascio prolungato - Nucleo: Lattosio, Polivinilpirrolidone, Ammoniometacrilato copolimero in dispersione, Triacetina, Alcool stearilico, Talco, Magnesio stearato, Rivestimento: Ipromellosa (E464), Titanio biossido (E171), Macrogol, Polisorbato 80, Ossido di ferro (E172). OxyContin 80 mg compresse a rilascio prolungato - Nucleo: Lattosio, Polivinilpirrolidone, Ammoniometacrilato copolimero in dispersione, Triacetina, Alcool stearilico, Talco, Magnesio stearato, Rivestimento: Ipromellosa (E464), Idrossipropilcellulosa, Titanio biossido (E171), Macrogol, Ossido di ferro (E172), Indaco carminio (E132).

**6.2 Incompatibilità.**

Non sono note incompatibilità

**6.3 Periodo di validità.**

36 mesi.

**6.4 Speciali precauzioni per la conservazione.**

Non conservare ad una temperatura superiore ai 25°C.

**6.5 Natura e capacità del contenitore.**

1) Contenitori in polipropilene con coperchi in polietilene (contenenti 28, 56 o 112 compresse). 2) Blister in PVC sostenuto con foglio di alluminio (contenenti 10, 28, 30, 56 o 112 compresse).

**6.6 Istruzioni per l’uso/manipolazione.** Nessuna.

**Dati amministrativi**

**7. TITOLARE DELL’AUTORIZZAZIONE ALL’IMMISSIONE IN COMMERCIO**

Mundipharma Pharmaceuticals Srl

Via G. Serbelloni, 4 - 20122 Milano

**8. NUMERO DELL’AUTORIZZAZIONE ALL’IMMISSIONE IN COMMERCIO**

OxyContin 10 mg compresse a rilascio prolungato – blister da 28 compresse:

AIC n° 034435014/M

OxyContin 10 mg compresse a rilascio prolungato – blister da 56 compresse:

AIC n° 034435026/M

OxyContin 10 mg compresse a rilascio prolungato – blister da 112

compresse: AIC n° 034435038/M

OxyContin 10 mg compresse a rilascio prolungato – flacone da 28

compresse: AIC n° 034435040/M

OxyContin 10 mg compresse a rilascio prolungato – flacone da 56

compresse: AIC n° 034435053/M

OxyContin 10 mg compresse a rilascio prolungato – flacone da 112

compresse: AIC n° 034435065/M

OxyContin 20 mg compresse a rilascio prolungato – blister da 28 compresse:

AIC n° 034435077/M

OxyContin 20 mg compresse a rilascio prolungato – blister da 56 compresse:

AIC n° 034435089/M

OxyContin 20 mg compresse a rilascio prolungato – blister da 112

compresse: AIC n° 034435091/M

OxyContin 20 mg compresse a rilascio prolungato – flacone da 28

compresse: AIC n° 034435103/M

OxyContin 20 mg compresse a rilascio prolungato – flacone da 56

compresse: AIC n° 034435115/M

OxyContin 20 mg compresse a rilascio prolungato – flacone da 112

compresse: AIC n° 034435127/M

OxyContin 40 mg compresse a rilascio prolungato – blister da 28 compresse:

AIC n° 034435139/M

OxyContin 40 mg compresse a rilascio prolungato – blister da 56 compresse:

AIC n° 034435141/M

OxyContin 40 mg compresse a rilascio prolungato – blister da 112

compresse: AIC n° 034435154/M

OxyContin 40 mg compresse a rilascio prolungato – flacone da 28

compresse: AIC n° 034435166/M

OxyContin 40 mg compresse a rilascio prolungato – flacone da 56

compresse: AIC n° 034435178/M

OxyContin 40 mg compresse a rilascio prolungato – flacone da 112

compresse: AIC n° 034435180/M

OxyContin 80 mg compresse a rilascio prolungato – blister da 28 compresse:

AIC n° 034435192/M

OxyContin 80 mg compresse a rilascio prolungato – blister da 56 compresse:

AIC n° 034435204/M

OxyContin 80 mg compresse a rilascio prolungato – blister da 112

compresse: AIC n° 034435216/M

OxyContin 80 mg compresse a rilascio prolungato – flacone da 28

compresse: AIC n° 034435228/M

OxyContin 80 mg compresse a rilascio prolungato – flacone da 56

compresse: AIC n° 034435230/M

OxyContin 80 mg compresse a rilascio prolungato – flacone da 112

compresse: AIC n° 034435242/M

**9. DATA DI PRIMA AUTORIZZAZIONE/RINNOVO AUTORIZZAZIONE**

Dicembre 2002

**10. TABELLA DI APPARTENENZA SECONDO IL D.P.R. 309/90**

Medicinale soggetto al DPR 309/90. Tabella IIª sez. A

**11. REGIME DI DISPENSAZIONE AL PUBBLICO**

Il prodotto deve essere venduto su presentazione di ricetta medica a ricalco.

Allegato Protocollo 4

Attestazioni di sicurezza dei componenti

Allegato Protocollo 5

Informazioni per il paziente

Questo modulo riporta le informazioni essenziali sullo studio clinico a cui Le viene proposto di partecipare. E’ importante che Lei legga queste informazioni e che le discuta con il Medico prima di firmare il consenso alla partecipazione allo studio (Modulo: “Consenso Informato alla Partecipazione allo Studio”). Anche dopo aver firmato, Lei può ritirare il Suo consenso alla partecipazione in ogni momento. Il rifiuto di partecipare o il ritiro del consenso non influirà in alcuno modo sulla cura che Lei riceve dai medici che La assistono

**Introduzione**

Questo studio clinico viene effettuato in pazienti con tumore e con dolore definito di tipo “neuropatico”. Questo tipo di dolore è di origine nervosa e può essere provocato da una compressione di nervi da parte del tumore.

In particolare i pazienti che partecipano a questo studio clinico hanno dolore con intensità moderata-severa.

Il dolore neuropatico è spesso difficile da trattare. Pertanto, il trattamento utilizzato in questi casi prevede in genere l'utilizzo di antidolorifici - oppioidi (simili alla morfina) deboli o forti, associati a farmaci adiuvanti. Questi ultimi non sono degli anti-dolorifici veri e propri ma sono farmaci in grado di modulare il grado di eccitamento nervoso, ottenendo come risultato la riduzione del dolore.

Il trattamento che si vuole valutare in questo studio clinico prevede l'utilizzo del farmaco antidolorifico ossicodone CR, associato al farmaco adiuvante pregabalin.

Entrambi questi farmaci hanno mostrato efficacia nel trattamento del dolore neuropatico. Tuttavia, mancano ancora informazioni circa il corretto modo di combinarli per ottimizzare i loro effetti e di ridurre i loro effetti collaterali.

**Obiettivi di questo studio clinico**

Questa ricerca si propone di studiare due possibili combinazioni di dosaggio dei farmaci ossicodone CR e pregabalin. In particolare lo studio valuterà l’efficacia e la tollerabilità delle due strategie di associazione.

Il raggiungimento dell’obiettivo si valuterà attraverso i seguenti parametri: percentuale di pazienti con riduzione di almeno un terzo dell’intensità del dolore, la velocità di raggiungimento del controllo del dolore e la presenza e il tipo di effetti collaterali.

Infine, una parte dello studio di ricerca cercherà di mostrare se alcune variabili genetiche dei pazienti sono responsabili di una differente risposta al trattamento con anti-dolorifici.

**Terapie nello studio**

Questo è uno studio di confronto tra due strategie di associazione dei farmaci ossicodone CR e pregabalin, che sono le seguenti:

- Strategia A: pazienti che assumono una dose fissa di ossicodone CR mentre la dose di pregabalin viene aumentata in modo graduale fino a ottenere un buon controllo del dolore osservando i livelli di efficacia e sicurezza dell’associazione
- Strategia B: pazienti che assumono una dose fissa di pregabalin mentre la dose di ossicodone CR viene aumentata in modo graduale fino a ottenere un buon controllo del dolore e osservando i livelli di efficacia e sicurezza dell’associazione

Nel caso di comparsa di dolore particolarmente fastidioso nel corso dello studio tutti i partecipanti potranno ricevere una terapia “di salvataggio” con morfina.

Per garantire l’obiettività dei risultati i partecipanti devono essere assegnati ai gruppi di trattamento in modo casuale, “randomizzato”, deciso da un computer.

**Procedure dello studio**

E’ previsto che partecipi a questo studio clinico un totale di 80 persone. L’inclusione dei partecipanti nello studio durerà un anno.

Se decide di partecipare allo studio Le verrà chiesto di firmare un consenso informato. Inoltre, Le verranno chieste delle informazioni di tipo socio-demografico (quali sesso, età, professione, istruzione).

Dopo l’inclusione nello studio, Lei verrà assegnato a uno dei due gruppi di trattamento sopra menzionati.

Lo schema di trattamento previsto dallo studio dura 14 giorni almeno. Questa durata è necessaria per poter valutare appieno gli effetti dei farmaci.

**L’organizzazione dello studio**

Lo studio prevede che i pazienti con dolore neuropatico vengano trattati con ossicodone, per 7 giorni, fino ad ottenere una stabilizzazione di trattamento e siano successivamente assegnati in modo casuale ai due gruppi che assumeranno ossicodone CR e pregabalin, a dosaggi diversi.

Lo studio dura 2 settimane e prevede 4 visite: una di inclusione, una di randomizzazione e 2 di controllo rispettivamente dopo 7 e 14 giorni dall'inizio del trattamento. In queste occasioni oltre alla consueta visita medica, verrà effettuata una dettagliata valutazione del dolore e degli effetti collaterali dei farmaci assunti. Inoltre, Lei sarà contattato/a telefonicamente in 2 occasioni: tre e dieci giorni dopo l’inizio del trattamento.

Infine, Le verrà chiesto di compilare un diario giornaliero annotando, tutti i giorni, le caratteristiche del dolore secondo un questionario predefinito e anche con parole Sue.

Il giorno dell’inizio del trattamento con ossicodone CR, se Lei non è in trattamento con tale farmaco, oppure il giorno in cui verrà assegnato a uno dei due gruppi, se lei è già in trattamento con ossicodone CR, sarà sottoposto a prelievo ematico per valutare alcuni parametri (emocromo e creatinina). Tale prelievo verrà ripetuto dopo 14 giorni.

**Studio sui polimorfismi genetici**

La ricerca scientifica negli ultimi anni ha acquisito nuove informazioni sui geni e, in particolare, sulle loro possibilità di influire sul nostro aspetto fisico (alcuni geni infatti controllano il colore degli occhi o dei capelli) e sulle condizioni di salute. Le ricerche, tendenti a stabilire le potenziali correlazioni-reazioni esistenti tra geni e buon esito di una terapia, effettuata con un dato farmaco, sono tuttavia ancora in fase iniziale.

In particolare la ricerca a cui le proponiamo di partecipare ha il fine di valutare il ruolo di alcuni fattori genetici nel predisporre il suo organismo a modificare la velocità con cui il farmaco viene metabolizzato e la sua attività. Tale ricerca ci serve per ottenere informazioni che potranno essere utili per individualizzare la terapia antidolorifica.

Lo sponsor della ricerca genetica è la Associazione Progetto Oncologia UMAN.A per la ricerca scientifica.

###### Procedure dello studio sui polimorfismi

Se acconsente a prendere parte a questo studio, le verrà prelevato un campione di sangue (circa 2 ml) da personale specializzato al momento della randomizzazione.

Dalle cellule del sangue prelevato verranno valutate alcune caratteristiche genetiche presenti sul DNA che potrebbero essere associate ad una diversa risposta ai farmaci antidolorifici che le vengono somministrati. Questa informazione permetterà di capire se è possibile individualizzare tipo e dosi di farmaci per il controllo ottimale del dolore in condizioni come la sua.

Al fine di salvaguardare la riservatezza, le informazioni personali e i campioni che La riguardano verranno indicati con un codice numerico, non con l’anagrafica personale. Solo il medico dello studio sarà in grado di associare il numero-soggetto ai diversi soggetti partecipanti alla ricerca.

La conservazione del campione sarà affidata al suo ospedale e presso gli istituti adibiti alla ricerca. I campioni eccedenti al termine della ricerca saranno distrutti secondo la normativa vigente.

**I vantaggi potenziali dello studio clinico.**

Benefici diretti: i partecipanti a questo studio potrebbero ottenere un buon bilancio fra efficacia analgesica ed effetti collaterali del loro trattamento antidolorifico.

Benefici per la comunità dei pazienti: lo studio potrebbe fornire indicazioni più precise sulle strategie terapeutiche da adottare nel caso di dolore di origine nervosa in pazienti con tumore.

**I rischi**

Il maggiore rischio legato alla partecipazione nello studio è rappresentato dagli effetti collaterali tipici dei farmaci. Questi disturbi sono sovrapponibili a quelli che si verificano quando i farmaci vengono assunti nella terapia del dolore.

I principali effetti collaterali sono:

Ossicodone CR: stitichezza, nausea, vomito, prurito, capogiri, sonnolenza.

La reazione avversa più grave, così come per tutti gli altri oppiacei, è la depressione respiratoria.

Pregabalin: capogiri, sonnolenza, aumento dell’appetito, vomito, secchezza delle mucose orali, stitichezza ed edema periferico. Si segnala, qualora il trattamento venga interrotto bruscamente, la possibile comparsa di sindrome da astinenza (insonnia, cefalea, diarrea).

**Controindicazioni ai farmaci in studio**

Entrambi i farmaci utilizzati nello studio sono controindicati in gravidanza e durante l'allattamento, in caso di alcune patologie respiratorie quali: ostruzione grave delle vie respiratorie, asma bronchiale, insufficienza respiratoria (ipercapnia) e depressione respiratoria, in patologie addominali compresi: ileo paralitico, addome acuto e svuotamento gastrico rallentato.

Altre controindicazioni all’uso dei farmaci sono: lesioni craniche, nota ipersensibilità a ossicodone, morfina o altri oppioidi, insufficienza epatica acuta, assunzione concomitante di IMAO, 24 ore prima e 24 ore dopo un intervento chirurgico.

Pertanto Lei dovrebbe comunicare al medico curante i trattamenti medici e chirurgici ai quali è sottoposto/a e la presenza di qualsiasi patologia medica di rilievo.

**I costi**

La partecipazione allo studio non Le comporterà costi aggiuntivi direttamente collegati alla partecipazione allo studio stesso

**La coperture assicurativa**

L’ospedale nel quale si svolge lo studio è assicurato per coprire eventuali danni derivanti dalla partecipazione a questo studio

**Confidenzialità**

Le chiediamo di autorizzarci, ai sensi della legge sulla privacy (DL n° 196 del 30 giugno 2003), a comunicare i dati personali che La riguardano al centro di coordinamento della ricerca. Il Suo nome, indirizzo e altri dati che la possono identificare saranno tolti da tutte le informazioni che Lei ci fornirà e saranno sostituiti con un codice numerico unico.

Il file con i nomi dei partecipanti e i codici numerici, sarà tenuto nel centro della ricerca in un computer protetto da una password. Le persone che avranno accesso a questo documento sono i Suoi medici curanti e il personale del centro di coordinamento della ricerca autorizzato per legge ad avere accesso ai dati, come statistici e persone incaricate della gestione dei dati.

I risultati dello studio saranno analizzati sotto forma di fenomeni generali, tabelle e grafici in modo che né Lei né altri partecipanti potrete essere mai identificati.

**Diritto di rifiutare o di interrompere lo Studio**

E’ importante che Lei sia consapevole del fatto che:

- la Sua decisione di partecipare a questo studio deve essere libera e presa sulla base delle informazioni contenute in questo modulo e di quelle che Lei vorrà chiedere al Suo medico;
- se Lei rifiuterà di partecipare a questo studio verrà comunque trattato/a presso questa struttura (se lo vorrà) e la qualità dell’assistenza che Le verrà fornita non sarà in alcun modo modificata;
- anche dopo avere dato il Suo consenso, Lei può in qualsiasi momento decidere di ritirarsi dallo studio, senza che ciò modifichi in alcun modo l’assistenza che riceverà

**Garanzie a tutela del partecipante allo studio**

- Questo studio di ricerca è stato approvato dal Comitato Etico Indipendente presso (da compilare) ………………………………………………………………………………………………………………………………………………………………………………………………………………………………………….………………………………………..

**Persone da contattare**

Se deciderà di aderire allo studio Le sarà consegnata una scheda con i recapiti dei medici responsabili clinici dello studio, per ogni informazione e necessità durante lo svolgimento dello studio stesso.

Inoltre, una copia di questa Scheda Informativa e una copia dell’eventuale Consenso resteranno in Suo possesso.

Allegato Protocollo 6

CONSENSO INFORMATO

***Studio di fase II randomizzato per valutare l’efficacia e la tollerabilità di***

***ossicodone CR a dosaggio fisso e pregabalin a dosi crescenti***

***versus ossicodone CR a dosi crescenti e pregabalin a dosaggio fisso***

***nel trattamento del dolore neuropatico in pazienti affetti da cancro***

Questo modulo deve essere firmato da Lei nel caso decida di partecipare allo studio clinico. E’ importante che Lei abbia discusso approfonditamente tutti gli aspetti dello studio con il Suo Medico prima di prendere la Sua decisione, anche sulla base della scheda riassuntiva a cui esso si riferisce (Modulo “Informazione per il Paziente”). Lei può ritirare il suo consenso in ogni momento.

**Io sottoscritto, nella mia piena capacità di intendere e di volere:**

**1) dichiaro di essere stato/a esaurientemente informato/a a proposito dello studio clinico in oggetto, di averne letto la Scheda Informativa per il Paziente di cui,**

**in data** …../…../………., **ho ricevuto una copia da conservare, e di averne discusso adeguatamente con i Medici che ne sono responsabili presso questa istituzione, in data** …../…../……… **presso** ………………...……………………………………………………..**;**

**2) dichiaro di essere consapevole, fra l’altro, dei vantaggi e dei rischi potenzialmente collegati all’oggetto dello studio, delle procedure dello studio, delle cautele che mi sono state richieste, e delle garanzie previste a mia tutela (come riportate dal modulo di Informazione per il Paziente);**

**3) rilascio il mio consenso libero e informato a prendere parte allo studio clinico in oggetto;**

**4) autorizzo, per gli scopi di questo studio clinico, il trattamento dei dati che mi riguardano, in formato cartaceo ed elettronico, nel rispetto delle normative vigenti, ai sensi, fra l’altro, del Decreto Lgs. 196/2003 (“Legge sulla privacy”) e successive modifiche, a integrazione del consenso da me già sottoscritto al trattamento dei dati sensibili presso l’A.O. Fatebenefratelli e Oftalmico;**

**5) autorizzo + non autorizzo +**

**a fornire al mio Medico di Medicina Generale,**

**dr.** ……………………………………………………………...………

**notizie sulla mia partecipazione a questo studio clinico.**

**Firma del Paziente**

………………………………….………

Nominativo ………………………………….……… Data: …../…../……….

**Firma del Rappresentante legale o del Tutore *(se appropriato)*** ………………………………….………

Nominativo ………………………………….……… Data: …../…../……….

**Firma di un Testimone** *(se appropriato)*

………………………………….………

Nominativo ………………………………….……… Data: …../…../……….

**Firma di un Testimone** *(se appropriato)*

………………………………….………

Nominativo ………………………………….……… Data: …../…../……….

**Firma del Ricercatore**

………………………………….………

Nominativo ………………………………….……… Data: …../…../……….

Allegato Protocollo 7

Modulo consenso

per lo studio sui fattori biologici

**Studio di fase II randomizzato per valutare l’efficacia e la tollerabilità di ossicodone CR a dosaggio fisso e pregabalin a dosi crescenti versus ossicodone CR a dosi crescenti e pregabalin a dosaggio fisso nel trattamento del dolore neuropatico in pazienti affetti da cancro**

Con la sottoscrizione del presente consenso non rinuncia a nessuno dei suoi diritti legali.

La firma attesta che:

1. Lei ha letto il modulo e ha ricevuto informazioni riguardo lo studio;
2. Lei ha discusso la ricerca con domande ed è soddisfatto delle risposte ricevute;
3. Lei ha avuto tempo per valutare l’eventuale partecipazione personale allo studio;
4. Lei accetta di partecipare a questa ricerca genetica.

Desidera che il medico di famiglia sia informato della sua partecipazione personale a questo studio genetico?

Sì **+** No **+**

Nome del paziente ……………………………………………………………………………

Firma del paziente ……………………………………………………………………………

Data ………/…………/…………

Rappresentante: terzo (testimone), familiare, legale rappresentante
(usare come richiesto a seconda del caso)

Nome ……………………………………………………………………………

Firma ……………………………………………………………………………

Data ………/…………/…………

Persona che riceve il consenso del paziente

Nome ……………………………………………………………………………

Firma ……………………………………………………………………………

Data ………/…………/…………

Acconsento che, al termine dello studio, il mio campione biologico venga impiegato per ulteriori studi tra caratteristiche genetiche e effetto dei farmaci antidolorifici

*Nome* ……………………………………..………………………………………

*Firma .......................................................................... Data ………/………/……....*

Allegato Protocollo 8

Informazioni per il medico di base

Egregio Dr,

il suo paziente ha recentemente acconsentito a prendere parte ad uno studio clinico disegnato per l’impiego di ossicodone CR, farmaco oppioide forte da utilizzarsi nel caso di dolore da moderato a grave che non risponde a FANS o ad oppioidi deboli (tramadolo, codeina) associato all’anticonvulsivante pregabalin.

Lo studio è di tipo randomizzato.

Lo studio prevede che i pazienti vengano arruolati in base a dei criteri di eleggibilità e riceveranno ossicodone CR e pregabalin, a dosaggi diversi, in base al gruppo in cui ogni singolo paziente verrà randomizzato. Il periodo di osservazione durerà 14 giorni. I pazienti verranno consultati e visitati più volte ed in occasione di ogni visita si valuteranno gli effetti collaterali, l’intensità del dolore e lo stato di salute generale.

È prevista anche una ricerca sull’associazione tra polimorfismi genetici e cinetica e dinamica del farmaci erogatiti Tali ricerca ci serve per ottenere informazioni che potranno essere utili per individualizzare la terapia antidolorifica.

Sarò regolarmente in contatto con il suo paziente durante lo studio, ma ciò nonostante le sarei molto grato se potesse contattarmi qualora il suo paziente dovesse riportarle qualsiasi nuovo sintomo o problema nei prossimi 14 giorni.

La informo che ho chiesto e ricevuto approvazione dal Comitato Etico per partecipare in qualità di Sperimentatore in questo studio.

La ringrazio in anticipo per la sua collaborazione in merito a questo studio.

Cordiali Saluti,
